# Insights into *Clematis cirrhosa* L. Ethanol Extract: Cytotoxic Effects, LC-ESI-QTOF-MS/MS Chemical Profiling, Molecular Docking, and Acute Toxicity Study

**DOI:** 10.3390/ph17101347

**Published:** 2024-10-09

**Authors:** Manal I. Alruwad, Riham Salah El Dine, Abdallah M. Gendy, Abdulrahman M. Saleh, Mohamed A. Khalaf, Hala M. El Hefnawy, Manal M. Sabry

**Affiliations:** 1 Department of Pharmacognosy, Faculty of Pharmacy, Cairo University, Kasr El-Aini Street, Cairo 11562, Egypt; manal.ib.ruwad@std.pharma.cu.edu.eg (M.I.A.); riham.salaheldine@pharma.cu.edu.eg (R.S.E.D.); hala.elhefnawy@pharma.cu.edu.eg (H.M.E.H.); manal.sabry@pharma.cu.edu.eg (M.M.S.); 2 Department of Pharmacology and Toxicology, Faculty of Pharmacy, October 6 University, Giza 12585, Egypt; 3 Department of Pharmaceutical Medicinal Chemistry and Drug Design, Faculty of Pharmacy (Boys), Al-Azhar University, Cairo 11884, Egypt; abdo.saleh240@azhar.edu.eg; 4 Department of Chemistry, College of Science, United Arab Emirates University, Al-Ain P.O. Box 15551, United Arab Emirates; 202170149@uaeu.ac.ae

**Keywords:** *Clematis cirrhosa*, antiproliferative assays, LC-ESI-QTOF-MS/MS, docking investigation, acute toxicity

## Abstract

Background: In Jordanian traditional medicine, *Clematis cirrhosa* is commonly employed for the management of different diseases. Numerous investigations have documented the cytotoxic properties of different *Clematis* species against numerous types of cancer. Previously, we demonstrated the potential cytotoxicity of *Clematis cirrhosa* against HT-29 colorectal cancer cells. Extending our work, the current research aimed to explore the possible mechanisms underlying its antiproliferative activity with a plant safety evaluation. Methods: This study evaluates the extract’s impact on the cell cycle, apoptosis, and cell migration through in vitro assays, LC-ESI-QTOF-MS/MS analysis, docking studies, and an acute toxicity evaluation. Results: The *Clematis cirrhosa* ethanol extract (CEE) induced G2/M phase cell cycle arrest (19.63%), triggered significant apoptosis (41.99%), and inhibited cell migration/wound healing by 28.15%. Quantitative reverse transcription polymerase chain reaction (qRT-PCR) analysis revealed increased expression of the proapoptotic markers BAX (6.03-fold) and caspase-3 (6.59-fold), along with the reduced expression of the antiapoptotic BCL-2, in CEE-treated cells. Moreover, CEE significantly restrained angiogenesis by reducing VEGF mRNA expression by 63.9%. High-resolution LC-ESI-QTOF-MS/MS studies identified 26 metabolites, including phenolic compounds, fatty acids, and triterpenoids. Docking studies suggested that manghaslin had the highest binding affinity for VEGFR-2, followed by calceolarioside B, quercetin 7-*O*-rhamnopyranoside, luteolin, and quercetin-3,7-*O*-diglucoside. On the other hand, salvadoraside exhibited the highest binding affinity for the inhibition of caspase-3, followed by quercetin-3,7-*O*-diglucoside, kaempferol-3,7-*O*-*α*-L-dirhamnoside, manghaslin, and tectoridin, supporting the observed apoptotic effects. Interestingly, the outcomes further indicate that a single oral administration of up to 5000 mg/kg CEE is safe for consumption. Conclusions: These outcomes point to the potential of *Clematis cirrhosa* as a promising candidate for further exploration in cancer therapy.

## 1. Introduction

Globally, cancer is a major contributor to mortality, causing nearly 10 million deaths yearly. This translates to approximately one out of every six deaths [1]. Although chemotherapy has shown advancements in the form of targeted therapy for cancer, it continues to be plagued by numerous unavoidable adverse effects and complications [2]. Medicinal plants have been considered for centuries as a valuable resource for various natural remedies. It is noteworthy that approximately one-quarter of the prescribed medications worldwide are derived from either wild or cultivated plants [3]. Plant secondary metabolites are being extensively studied for their potential antitumor activity, leading to the development of innovative clinical drugs [4]. Key plant-derived products include vinca alkaloids like vincristine [5] and vinblastine [6] from *Catharanthus roseus*, paclitaxel from *Taxus baccata* [7], podophyllotoxin from *Podophyllum peltatum* [8], and camptothecin derivatives like topotecan and irinotecan from *Camptotheca acuminate* [9]. *Clematis*, a genus belonging to the Ranunculaceae family, is widely regarded as one of the most representative genera. This cosmopolitan genus is native in the temperate zones of both hemispheres but with some species distributed in tropical areas; this genus boasts around 350 species [10]. *Clematis* has been identified as a botanical source of miscellaneous groups of secondary metabolites. These include flavonoids, phenolic glycosides, lignans, coumarins, fatty acids, macrocyclic compounds, steroids, triterpenes, and volatile oils [11]. This diversity of secondary metabolites found in *Clematis* species is indicative of their various types of biological activity. *Clematis* species exhibit notable biological effects, mainly antinociceptive, anti-inflammatory, antitumoral, antioxidant, and antibacterial properties [12,13,14]. Many *Clematis* species are used to cure different ailments in traditional herbal medicine, such as rheumatic conditions, eye infections, fever, and dermatological problems. Additionally, *Clematis* is known for its potential as a remedy for snake bites and its possible antimalarial properties [15]. In Jordan, two species of *Clematis*—specifically, *C. cirrhosa* L. and *C. flammula* L.—are present [16]. In Jordanian traditional medicine, *C. cirrhosa* is commonly employed for the management of skin diseases [17,18]. Numerous investigations have documented the cytotoxic properties of the *Clematis* genus against numerous types of cancer. *C. flammula* extract showed significant cytotoxicity against Chinese hamster lung (CHL) and human liver (HL) cancer cell lines [19]. Saponins derived from *C. argentilucida* roots and *C. tangutica* [14] exhibited potent cytotoxic effects against human liver (HepG2), human leukemia (HL-60), and human glioblastoma multiforme (U251MG) cancer cell lines, outperforming doxorubicin (DOX) and nimustine [20].

Based on our previous study demonstrating the cytotoxic effects of *Clematis cirrhosa* against HT-29 colorectal cancer cells [21], the current research aimed to further tackle the possible mechanisms underlying the antiproliferative activity of the *Clematis cirrhosa* ethanol extract (CEE) against HT-29 cells by evaluating its impact on the cell cycle, apoptosis, and cell migration.Additionally, it aimed to preliminarily investigate the bioactive compounds present in an extract obtained from *C. cirrhosa* utilizing high-resolution LC-ESI-QTOF-MS/MS spectrometry. Furthermore, the effects of CEE active metabolites on the vascular endothelial growth factor 2 tyrosine kinase receptor (VEGFR-2 TK) and caspase-3 proteins were evaluated via molecular docking studies. Finally, the acute toxicity associated with asingle oral administration of CEE was determined.

## 2. Results

### 2.1. Cytotoxicity Evaluation

#### 2.1.1. Cell Cycle Analysis

**Figure 1:**
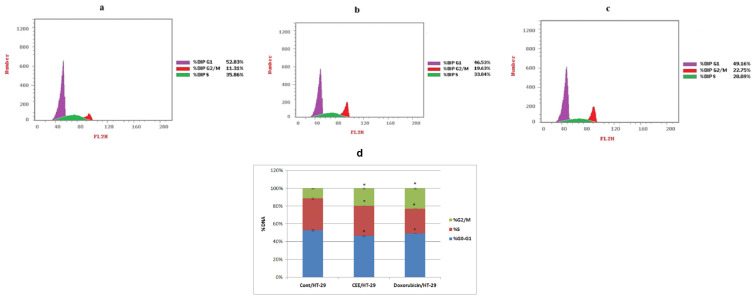
Cell cycle analysis for HT-29 cells treated with IC_50_ of CEE and DOX for 72 h. (**a**) The control consisted of HT-29untreated cells; (**b**) HT-29 cells were treated with CEE; (**c**) HT-29 cells were treated with DOX. (**d**) Comparative analysis of the sub-G0/G1, S, and G2/M phases across different groups. * represents a significant result (*p* < 0.05) compared with untreated cells.

The results, illustrated in Figure 1, demonstrated significant changes in the distribution of distinct cell cycle phases upon treatment with either CEE or doxorubicin (DOX). Both CEE and DOX treatments led to distinct G2/M phase cell cycle arrest, with percentages of 19.63% and 22.75%, respectively, compared to 11.31% in the control. The pronounced antiproliferative effectsof CEE in HT-29 cells are potentially mediated by cell cycle regulation.

#### 2.1.2. Flow Cytometry-Based Annexin V-FITC/PI Assay for the Detection of Apoptosis

**Figure 2:**
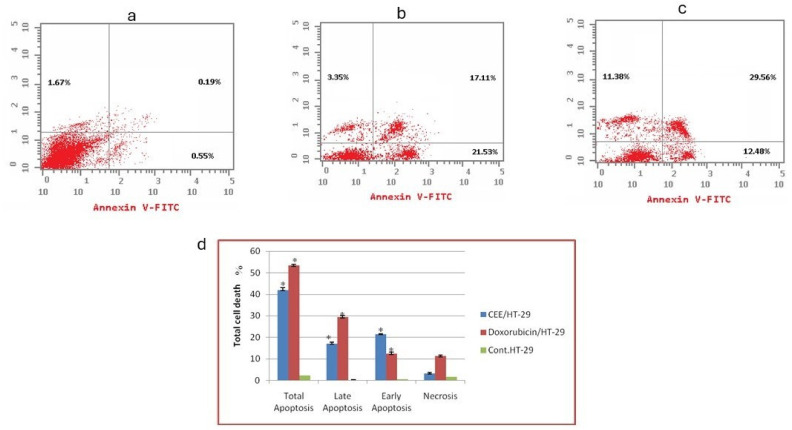
The effects of *Clematis cirrhosa* ethanol extract CEE and DOX on apoptosis and necrosis in HT-29 cells. (**a**) The control consisted of untreated cells of HT-29; (**b**) HT-29 cells were treated with CEE; (**c**) HT-29 cells were treated with DOX. (**d**) Comparative analysis of early and total apoptosis for HT-29. * indicates a significant result compared with untreated cells (*p* < 0.05).

This assay allows the differentiation of apoptotic cells (detected by Annexin V labeling) from living cells, while necrotic cells can be identified by PI staining. In the non-treated group, the total percentage of HT-29 cell death was 2.41% (Figure 2a). However, treatment with CEE resulted in significantly higher total cell death of 41.99% in HT-29 cells (Figure 2b). These findings are aligned with the previous Sulforhodamine B (SRB) assay results [21]. Similarly, DOX treatment induced total cell death of 53.42% in HT-29 cells (Figure 2c). By comparing CEE with DOX, the primary mechanism of cell death induced by CEE against HT-29 cells was found to be predominantly apoptotic rather than necrotic, emphasizing the cytotoxic effect of CEE through apoptotic pathways.

#### 2.1.3. Scratch Wound Healing

**Figure 3:**
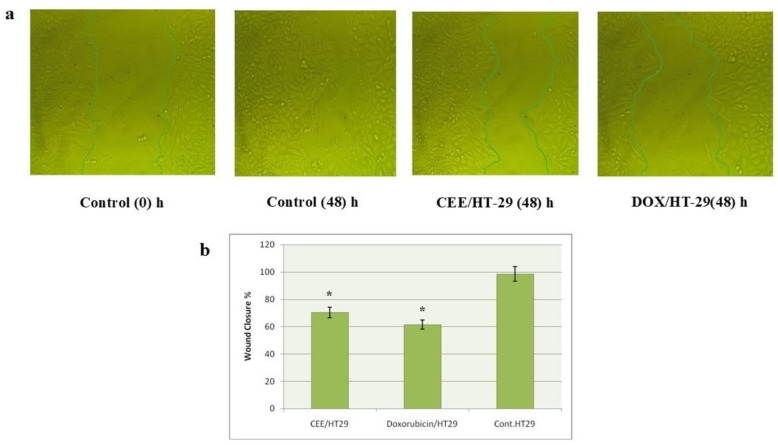
(**a**) Effect of CEE and DOX on cell migration in HT-29 cells. Scratching was performed with a 1 mL pipette tip; (**b**) quantitative representation of the migration of HT-29 in the scratch wound healing assay. Data are presented as the mean and SD. * indicates a significant result compared with untreated cells (*p* < 0.05).

The impact of CEE and DOX on the cell growth and migratory behavior of HT-29 cells was examined. In the control group, the scratched area of the cell monolayer was completely healed after 48 h. However, the IC_50_ concentration of CEE induced a notable delay in the closure of the scratched area of HT-29 cells in comparison to both untreated cells and cells treated with DOX. Specifically, CEE treatment led to a 28.15% decrease in wound closure in HT-29 cells compared to the control group. Similarly, DOX treatment resulted in a 37.04% decrease in closure compared to the control group (Figure 3a).

#### 2.1.4. mRNA Transcription Levels of VEGF, BAX, BCL-2, and Caspase-3

**Figure 4:**
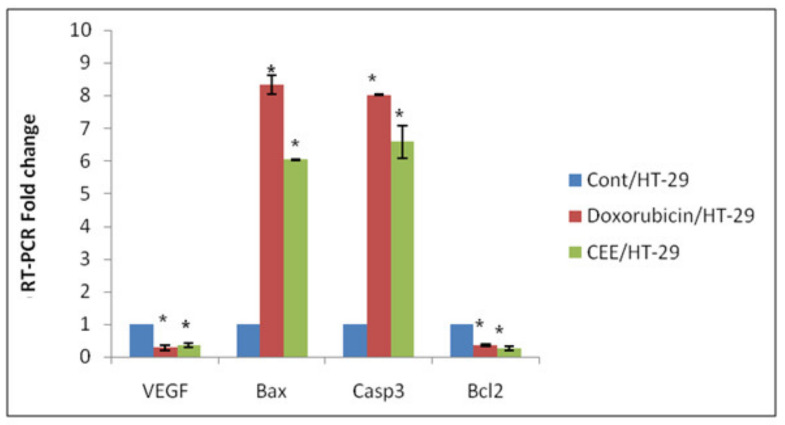
Gene transcription levels of VEGF, BAX, BCL-2, and caspase-3. HT-29 cells treated with the CEE or DOX standard at 72 h. Data are presented as the mean ± SD. * represents a significant result at *p* < 0.05 compared with untreated cells.

To investigate the patterns of molecular transcription associated with the healing of wounds, the levels of VEGF mRNA, an angiogenic factor, were assessed. As revealed in Figure 4, CEE treatment resulted in a significant reduction in VEGF mRNA expression by 63.9% in HT-29 cells in comparison to the untreated group. Notably, the impact of DOX was even more pronounced, with a 71.62% decrease in VEGF mRNA expression.

The mRNA transcription levels of apoptosis-regulating proteins, such as a proapoptotic member of the BCL-2 gene family (BAX), B-cell lymphoma 2 (BCL-2), and caspase-3, were investigated to investigate the underlying mechanism of apoptosis induction in HT-29 cells. In colorectal cancer cells treated with CEE, there was a substantial elevation in the mRNA levels of either BAX or caspase-3, with a fold increase of 6.03 and 6.59, respectively, compared to the untreated group. Similarly, in HT-29 cells treated with DOX, there was a spike in both BAX and caspase-3 mRNA levels, with fold increases of 8.33 and 8.02, respectively, compared to the untreated group. Conversely, the BCL-2 mRNA transcription level was significantly suppressed by the CEE and DOX treatments (0.2647- and 0.3542-fold change, respectively).

### 2.2. LC-ESI-TOF-MS/MS

**Figure 5:**
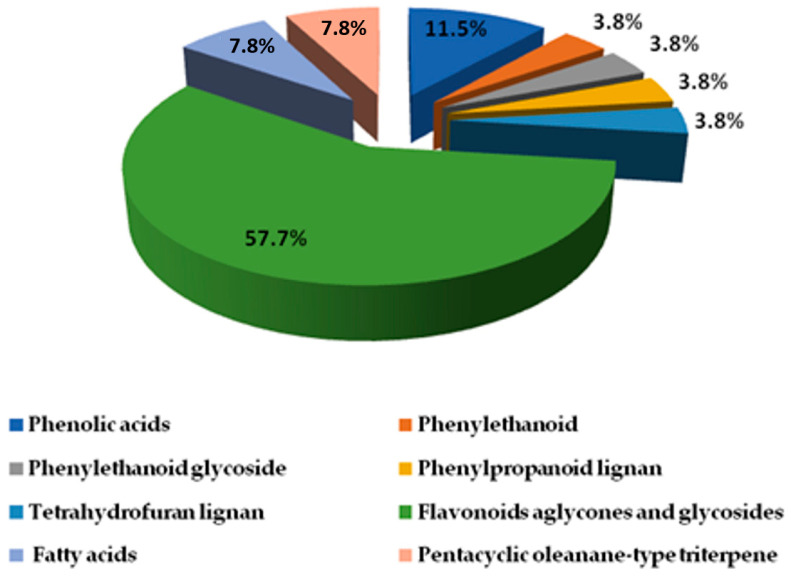
Phytochemical composition of *Clematis cirrhosa* extract determined using high-resolution LC-ESI-TOF-MS/MS spectrometry.

Analyzing the mass spectrometry (MS) data, MS/MS fragmentation information, literature reports, and fragmentation patterns, 26 metabolites were identified in the extract of *Clematis cirrhosa* (Figures S1 and S2). These compounds were classified into three categories, which included phenolic compounds, including three phenolic acids, one phenylethanoid, one phenylethanoid glycoside, one phenylpropanoid lignan, and one tetrahydrofuran lignan; 15 flavonoids, aglycones, and glycosides; two fatty acids; and two triterpenoids. Figure 5 illustrates the phytochemical composition of *C. cirrhosa*. Table 1 and Table 2 display the retention time, mass spectrometry information, and relevant literature associated with each of the identified compounds.

#### 2.2.1. Phenolic Compound Identification

##### Phenolic Acids, Phenylethanoid, and Phenylpropanoid

Three phenolic compounds have been identified in *C. cirrhosa*, including three hydroxycinnamic acids (caffeic acid, *p*-coumaric acid, and ferulic acid). Caffeic acid was determined to be the compound corresponding to peak (**4**, Figure S6). Its [M+H]^+^ was identified at *m*/*z* 181.0505. Further fragmentation led to the elimination of a water molecule and the generation of a distinctive ion at *m*/*z* 163 as [M+H-H_2_O]^+^ [43]. *p*-Coumaric acid (**6**, Figure S8) has a deprotonated molecular ion at *m*/*z* 163.0402 [M-H]^−^. Its MS/MS fragmentation pattern has been reported to typically involve the loss of a neutral molecule of carbon dioxide, with the resulting fragment ion at *m*/*z* 119, and it corresponds to the [M-H-CO_2_]^−^ [26]. Peak (**16**, Figure S18) was identified as ferulic acid. Its molecular ion was observed at *m*/*z* 193.0496 as [M-H]^−^, while the major fragment ion was observed at *m*/*z* 134 as [M-H-CO_2_-CH_3_]^−^ due to the loss of carbon dioxide and a methyl group [35]. In addition, one phenylethanoid was identified as 3,4-dihydroxyphenylethanol (hydroxytyrosol) for peak (**2**, Figure S4). A parent ion [M-H]^−^ was observed at *m*/*z* 153, and a fragment ion at *m*/*z* 123 as [M-H-CH_2_O]^−^ corresponded to the loss of formaldehyde [23]. Calceolarioside B (**3**, Figure S5) is a phenylpropanoid glycoside. Its molecular ion [M-H]^−^ was observed at *m*/*z* 477.1614. The mass spectrometry analysis showed two distinct product ions: one at *m*/*z* 161, corresponding to a caffeic acid moiety (C_9_H_5_O_3_), and another at *m*/*z* 315 (C_14_H_19_O_8_), representing the hydroxytyrosol glucoside part. This fragmentation pattern is consistent with the typical fragmentation patterns observed in phenylethanoid glycosides [24].

##### Lignans

**Figure 6:**
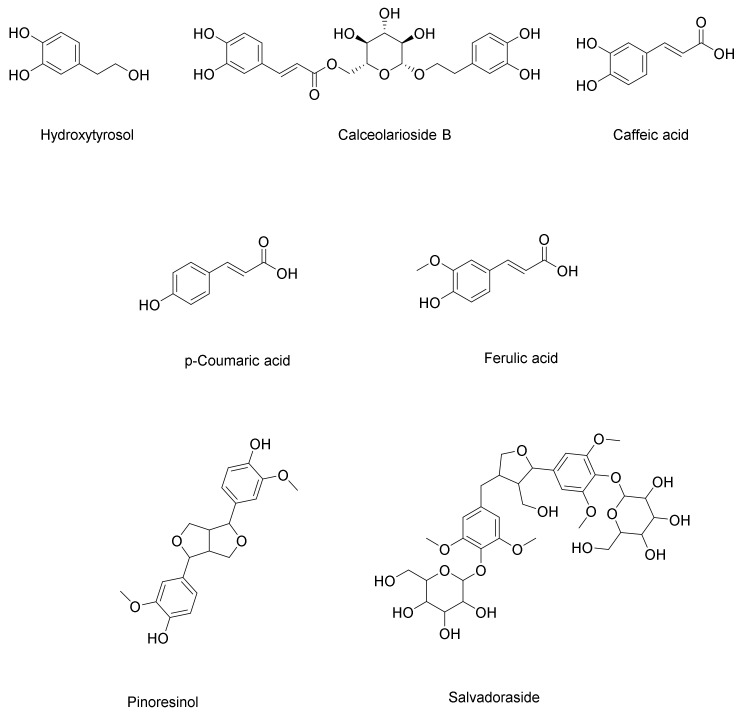
Chemical structures of phenolic acid, phenylethanoid, and lignans identified in *Clematis cirrhosa*.

*C. cirrhosa* was found to contain two types of lignans (Figure 6): a phenylpropanoid lignan called salvadoraside (peak **9**, Figure S11) and a tetrahydrofuran lignan known as pinoresinol (peak **10**, Figure S12). Phenolic lignans of the furofurano class involve cleavage between the α- and *β*-positions in the side chain, yielding product ions at *m*/*z* 151 (4-formyl-2-methoxyphenolate moiety/guaiacyl) or *m*/*z* 181 (syringyl), which is a highly distinctive pattern for this particular class of lignans and is considered to be the most prominent fragmentation pattern associated with their analysis using mass spectrometry in negative ion mode [30].

**Figure 7:**
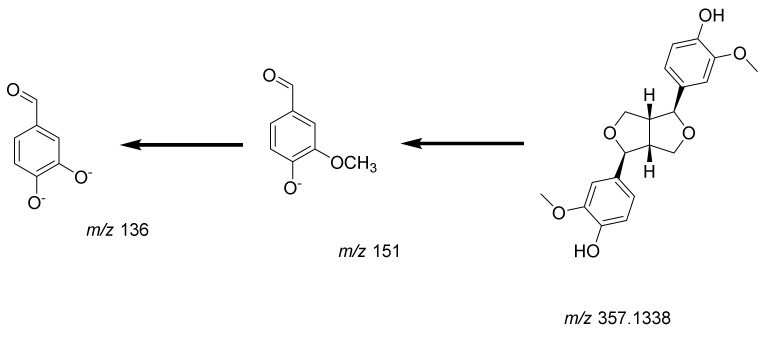
Fragmentation pattern of pinoresinol.

**Figure 8:**
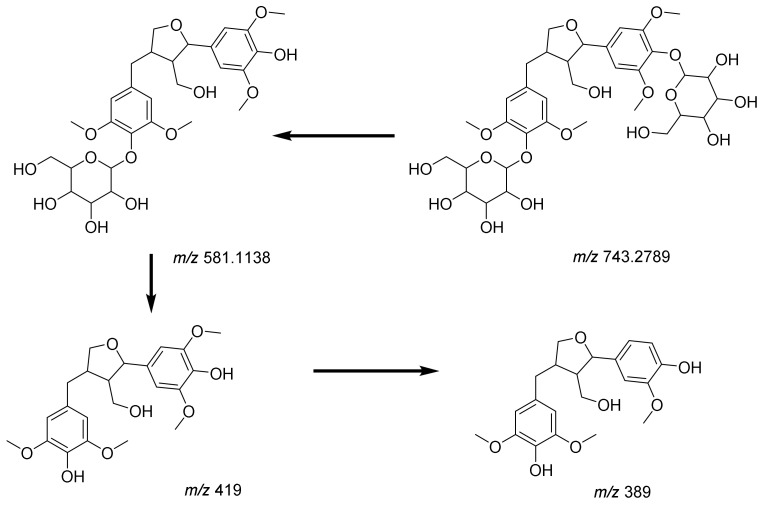
Fragmentation pattern of salvadoraside.

Pinoresinol (**10**, Figure S12 was identified at *m*/*z* 357.1338 as [M-H]^−^, showing two characteristic fragment ions at *m*/*z* 151 and *m*/*z* 136 as [M-H-C_8_H_7_O_3_-CH_3_]^−^, indicating the elimination of the4-formyl-2-methoxyphenolate moiety (C_8_H_7_O_3_) and methyl moiety, respectively [30] Figure 7 illustrates the fragmentation pattern of pinoresinol. Salvadoraside (**9**), a glycoside of geniposidic acid and previously isolated from *C. armandii* [15] exhibited a deprotonated molecular ion [M-H]^−^ at *m*/*z* 743.2789 and three product ions at *m*/*z* 581.1138 and 419 indicating the loss of two successive hexose moieties [M-H-C_6_H_12_O_6_]^−^ and [M-H–2(C_6_H_12_O_6_)]^−^, in addition to a fragment ion at *m*/*z* 389 indicating the loss of an aldehyde moiety [M-H-2(C6H_12_O_6_)–CH_2_O]^−^ [29]. Figure 8 illustrates the fragmentation pattern of salvadoraside. The structures of the identified phenolic acids, phenylethanoid, and lignan compounds are illustrated in Figure 6.

#### 2.2.2. Identification of Flavonoids (Aglycones and Glycosides)

This study revealed that *C. cirrhosa* contains a diverse range of flavonoids, including flavones such as acacetin and luteolin, flavanols like catechin, flavonols such as quercetin, and isoflavones like genistein and puerarin. Additionally, some of the identified flavonoids exist as glycosides, specifically *O*-glycosides and *C*-glycosides. Catechin (**5**, Figure S7) exhibited a deprotonated molecule [M-H]^−^ with an *m*/*z* value of 289.0713. Furthermore, two product ions at *m*/*z* of 245 and 205 corresponded to the removal of CO_2_ and the Aring, respectively [25]. In negative ionization mode, the peak (**18**, Figure S20) at *m*/*z* 269.0454 in the spectrum corresponded to genistein, an isoflavonoid compound. This identification is reinforced by the existence of specific product ions at *m*/*z* of 241 [M-H-CO]^−^, 225 [M-H-CO_2_]^−^, 197 [M-H-CO_2_-CO]^−^, and 143 [M-H-C_6_H_6_O_3_]^−^corresponding to cleavage at [0,4B] ^−^ and the formation of the [0,4B-H_2_O]^−^ ion [36]. Quercetin (**19**, Figure S21) had the [M-H]^−^ ion at *m*/*z* 301.0355. A product ion at *m*/*z* 273 as [M-H-CO]^−^ was found by MS/MS fragmentation analysis, and it represents the elimination of a carbon monoxide molecule. Furthermore, as a result of the RDA reaction, a production was formed at *m*/*z* 179, [1,3A]^−^ ions at *m*/*z* 151, and [0,4A]^−^ at *m*/*z* 107.01, which is indicative of ring A [37]. In positive mode, luteolin (**20**, Figure S22) was identified at *m*/*z* 285.0417 as [M-H]^−^, with subsequent fragmentations at *m*/*z* 241 as [M-H-CO_2_]^−^, indicating the loss of carbon dioxide from the C ring, and two fragments in the A ring at *m*/*z* 217 and 175, indicating the neutral loss of C_3_O_2_ and C_2_H_2_O, respectively. The prevailing product ions observed from the RDA fragmentation of luteolin were the 1,3B^−^ ion at *m*/*z* 133 and the 1,3A^−^ ion at *m*/*z* 151 [39]. Acacetin (24, Figure S26) was identified at *m*/*z* 285 as [M+H]^+^, showing two product ions at *m*/*z* 270 as [M+H-CH3]^+^ and 242 [M+H-CH3-CO]+, indicating the elimination of a methyl group followed by carbon monoxide. Further RDA fragmentation resulted in the formation of two ions, the 1,3B^−^ ion at *m*/*z* 133 and the 1,3A^−^ ion at *m*/*z* 153 [45].

**Figure 9:**
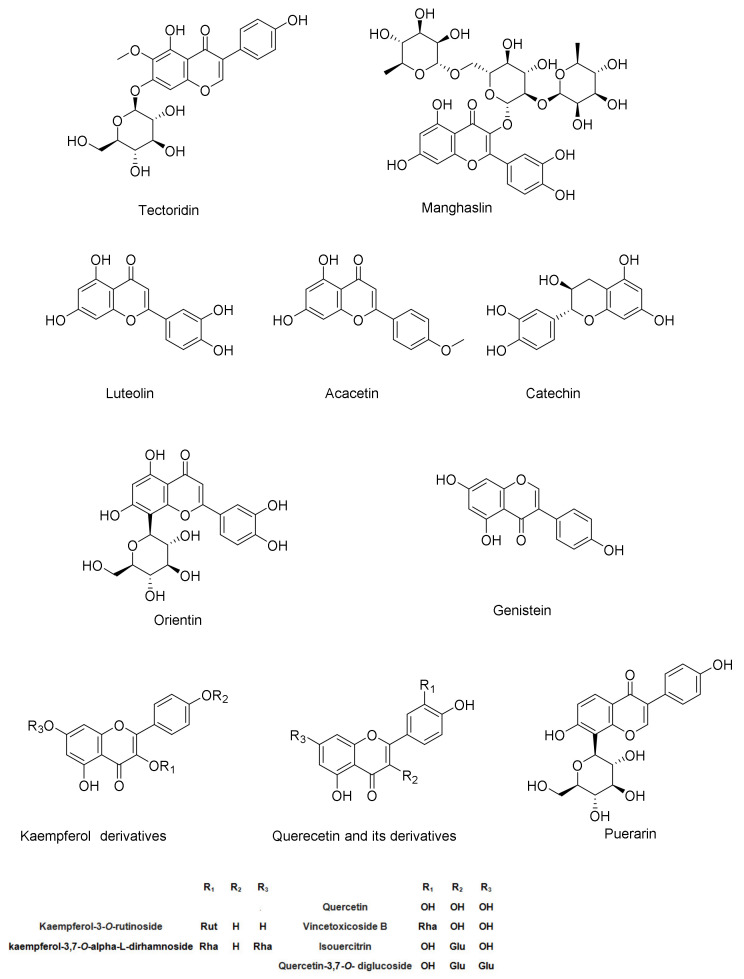
Chemical structures of flavonoids identified in *Clematis cirrhosa*.

A total of ten flavonoid glycosides were detected in *C. cirrhosa*, corresponding to peaks [**1**, **7**, **8**, **11**, **12**, **13**, **14**, **15**, **17**, and **21**] Chrysoeriol-*O*-hexoside (**1**, Figure S3) exhibited the [M-H]^−^ ion at *m*/*z* 461.1058, showing many fragmentions at *m*/*z* 446, which is equivalent to [M-H-CH_3_]^−^; at *m*/*z* 299 as [M-H-C_6_H_12_O_5_]^−^, corresponding to chrysoeriol aglycone; and at *m*/*z* 284 as [M-H-C_6_H_12_O_5_-CH_3_]^−^ [22]. Peak (**7**) was recognized with [M-H]^−^ at *m*/*z* 625.1409 and was identified as quercetin with two hexosyl residues. The fragment ion at *m*/*z* of 463 as [M-H-C_6_H_12_O_5_]^−^ corresponded to the elimination of a hexosyl moiety, and a fragment ion with *m*/*z* of 301 [M-H-2C_6_H_12_O_5_]^−^ corresponded to the elimination of both hexosyl residues. The fragment profile indicated that each hexosyl moiety was located ata different position in the aglycone. Based on this, peak (**7**, Figure S9) was recognized as quercetin-3,7-*O*-diglucoside [27]. Manghaslin (**8**, Figure S10), identified as [M−H]^−^ at *m*/*z* 755.2126, was found to be a form of quercetin 3-*O*-(2′-rhamnosyl)-rutinoside. The ion at *m*/*z* 591 as [M-H-C_6_H_12_O_5_]^−^ corresponds to the detachment of the rhamnosyl moiety from the molecular ion. Meanwhile, the presence of an ion at *m*/*z* 301, denoted as [M-H-C_18_H_31_O_13_]^−^, signifies the formation of the quercetin aglycone moiety through the additional elimination of the rutinosyl moiety [28]. Vincetoxicoside B (**11**, Figure S13), also known as quercetin-*O*-rhamnoside, was identified as [M-H]^−^ at *m*/*z* 447.0912. A product ion with *m*/*z* of 301 as [M-H-C_6_H_11_O_4_]^−^ corresponds to the elimination of the rhamnose moiety [31]. Peak (**12**, Figure S14) in the mass spectrum corresponds to luteolin monohexoside, showing the [M-H]^−^ ion at *m*/*z* 447.098, and three product ions were observed. The ion at *m*/*z* 285, as [M-H-C_6_H_12_O_5_]^−^, represents the detachment of a hexose moiety from the parent molecule. Two product ions were detected: at m/z 357, we observed [M-H-C_3_H_6_O_3_]^−^, which exhibited higher abundance, and, at *m*/*z* 327,we observed [M-H-C_4_H_8_O_4_]^−^, which was a result of the collision-induced dissociation of the aglycone at the 0–3 bond and the 0–2 bond, respectively. Based on these observations, the peak was identified as orientin, which is a flavone-*C*-glycoside containing a hexose moiety at the C-8 position [32]. Kaempferol-3,7-*O*-dirhamnoside (13, Figure S15) was identified at *m*/*z* 577.1563 as [M-H]^−^, along with two product ions. The first had *m*/*z* of 431 [M−H−C_6_H_11_O_4_]^−^, which correlated to the elimination of one rhamnose sugar moiety, while the second had *m*/*z* of 285 [M-H-2(C_6_H_11_O_4_)]^−^, which correlated to the elimination of both rhamnose sugar molecules from the kaempferol glycoside [33]. In the negative mode, the mass spectrum of isoquercitrin (**14**, Figure S16) was observed at *m*/*z* 463.0869 as [M-H]^−^. The prominent fragment ion at *m*/*z* 301, [M-H-C_6_H_12_O_5_]^−^, correlates to the elimination of a hexose moiety. Furthermore, a fragment ion at *m*/*z* 151, which correlated to [M-H-C_6_H_12_O_6_-H_2_O-C_8_H_8_O_2_]^−^, was detected. This fragment ion is formed through a retro-Diels–Alder rearrangement [47]. In the mass spectrum, kaempferol-3-*O*-rutinoside (**15**, Figure S17) was detected at *m*/*z* 593.150 as [M-H]^−^. Additionally, a prominent ion at *m*/*z* 285, represented as [M-H-C_12_H_22_O_10_]^−^, corresponds to the kaempferol aglycone resulting from the elimination of the rutinose moiety from the parent molecule [34]. Puerarin, a *C*-glycoside isoflavonoid of daidzein (**17**, Figure S19), exhibited a quasimolecular ion at *m*/*z* 417.121 [M+H]^+^. The fragmentation of puerarin can occur through various pathways, such as through the loss of a H_2_O molecule and cross-ring cleavages in the sugar moiety. The MS/MS spectra of mono-*C*-glycosides clearly distinguish them from *O*-glycosides. Aglycone product ions were not detected in the MS/MS spectrum; however, product ions from the cleavage of the sugar moiety were present [39]. Among the resultant fragment ions were *m*/*z* 399 [M+H-H_2_O]^+^, which was associated with the removal of an H_2_O molecule, and *m*/*z* 351 [M+H-2H_2_O-CH_2_O]^+^, indicating the elimination of CH_2_O, followed by *m*/*z* 267 as [M+H-2H_2_O-CH_2_O-C_4_H_4_O_2_]^+^ and *m*/*z* 297 as [M+H-C_4_H_8_O_4_]^+^ [41]. Tectoridin (**21**, Figure S23) was identified at *m*/*z* 463.133 as [M+H]^+^, exhibiting fragment ions at *m*/*z* 298.0472 [M+H-C_6_H_12_O_5_]^+^ and *m*/*z* 284.0317 [M+H-C_6_H_12_O_5_-CH_3_]^+^, indicating the molecular ion’s detachment of a hexose moiety followed by a methyl group [44]. The structures of the identified flavonoids are illustrated in Figure 9.

#### 2.2.3. Identification of Miscellaneous Compounds

**Figure 10:**
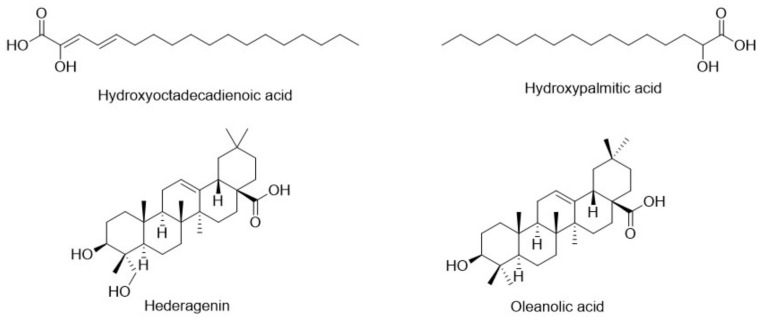
Chemical structures of fatty acids and triterpenoid identified in *Clematis cirrhosa*.

Two fatty acids, hydroxy octadecadienoic acid and hydroxy palmitic acid, as well as two pentacyclic oleanane-type triterpenoids, hederagenin (Figure S25) and oleanolic acid (Figure S28), were identified in *C. cirrhosa*. Hydroxy octadecadienoic acid (**22**, Figure S24) was identified in negative ionization mode at *m*/*z* 295.2266 as [M-H]^−^. Subsequently, two product ions were generated, at an *m*/*z* ratio of 277 as [M-H-H_2_O]^−^, correlating to the elimination of a water molecule, and an *m*/*z* ratio of 171, corresponding to a fragment with the formula C_9_H_15_O_3_ [39]. Hederagenin (**23**, Figure S25) has been identified based on the molecular ion with *m*/*z* of 471.348 [M-H]^−^ and exhibited fragmentation, with *m*/*z* values of 405 as [M-H-H_2_O-HCOOH]^−^ and 393 as [M-H-2H_2_O-HCOOH]^−^ [40]. Hydroxy palmitic acid (**25**, Figure S27) was detected at *m*/*z* 271.2274 as [M-H]^−^ and showed two product ions with *m*/*z* ratios of 253 as [M-H-H_2_O]^−^ and *m*/*z* 225 as [M-H-COOH]^−^ [39]. For oleanolic acid (**26**, Figure S28), the molecular ion peak was observed at *m*/*z* 455.3531 as [M–H]^−^. The obtained fragment ion was found at *m*/*z* 407 as [M-HCHO-H_2_O–H]^−^ [42]. Oleanolic acid and hederagenin-based triterpenoid glycosides have been identified as bioactive compounds isolated from *C. ganpiniana* [48]. The structures of the identified fatty acids and triterpenoids are illustrated in Figure 10.

### 2.3. Docking of Identified Metabolites in Extract of Clematis cirrhosa

The ethanol extract of *C. cirrhosa* exhibited potent activity against HT-29 cancer cells. The *in silico* molecular docking of the extracted metabolites and sorafenib, a positive control, with VEGFR-2 and caspase-3 confirmed their antitumor mechanisms.

#### 2.3.1. VEGFR-2 Inhibition

**Figure 11:**
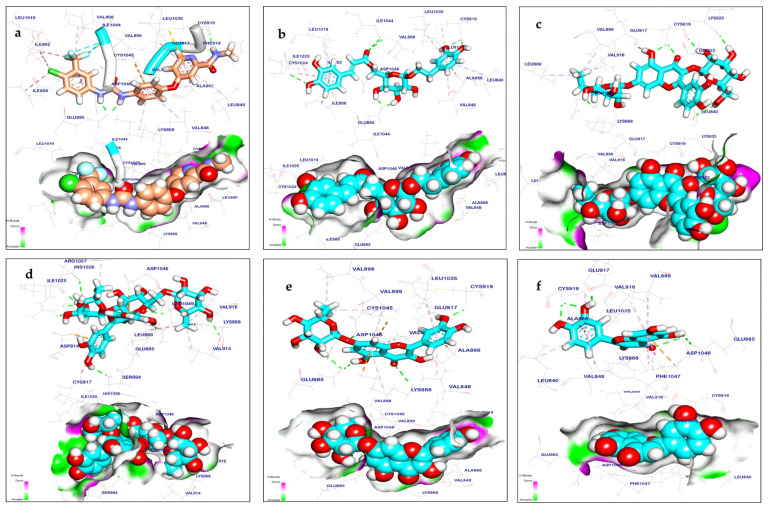
Mapping surfaces and 3D orientation of compounds docked inVEGFR-2 TK: (**a**) sorafenib; (**b**) calceolarioside; (**c**) quercetin-3,7-*O*-diglucoside; (**d**) manghasin; (**e**) quercetin 7-*O*-alpha-rhamnopyranoside; (**f**) luteolin.

Based on the docking data, the binding mechanism of the sorafenib ligand with VEGFR tyrosine kinase exhibited an energy binding score of −8.50 kcal/mol^−1^. It created nineteen hydrophobic interactions with Ala866, Leu840, Leu1025, Phe918 Val916, Lys868, Cys1045, Val848, Val899, Ala866, Leu1019, Ile892, Leu889, Ile888, and Ile1044; moreover, sorafenib exhibited five hydrogen bonds with Cys919, Asp1046, Glu885, and Val899 (Figure 11a). Calceolarioside B demonstrated energy binding of −9.20 kcal/mol^−1^. It formed nine hydrogen bond interactions with Val899, Ile1044, Ile1025, Asp1046, Glu885, Glu917, and Cys919, as well as nine hydrophobic interactions with Leu1019, Cys1024, Ile892, Ile888, Leu840, Cys919, Leu1035, Val848, and Ala866 (Figure 11b). Furthermore, quercetin-3,7-*O*-diglucoside’s binding mechanism revealed energy binding of −6.44 kcal/mol^−1^. Quercetin-3,7-*O*-diglucoside formed eight hydrogen bonds with Leu840, Lys920, Cys919, Glu917, and Asp1046 and three hydrophobic interactions with Val916, Leu889, and Val899 (Figure 11c). Manghaslin demonstrated energy binding of −9.68 kcal/mol^−1^. It formed eight hydrogen bonds with Val914, Ser884, Glu885, Ile1025, Lys868, Arg1027, and His1026, as well as nine hydrophobic interactions with Asp814, Asp1046, Leu1049, Cys817, Leu889, Val914, Lys868, and Val916 (Figure 11d). The binding energy of quercetin 7-*O*-rhamnopyranoside was found to be −8.37 kcal/mol^−1^. It formed six hydrogen bond interactions with Lys868, Cys919, Glu917, Glu885, and Asp1046 and nine hydrophobic interactions with Leu1035, Ala866, Cys1045, Val848, Val899, Val898, and Val916 (Figure 11e). The binding mechanism of luteolin revealed binding energy of −7.18 kcal/mol^−1^. It formed six hydrogen bond interactions with Asp1046, Cys919, Glu917, and Glu885 and also showed eight hydrophobic interactions with Leu1035, Lys868, Ala866, Val848, Cys919, Val899, Leu840, and Val916 (Figure 11f). Table 3 shows the free binding energy (kcal/mol) of the identified metabolites by LC-ESI-QTOF-MS/MS in terms of interactions with the target region of VEGFR-2 TK. Polymerase chain reaction (PDB) ID: 4ASD.

#### 2.3.2. Caspase-3 Inhibition

**Figure 12:**
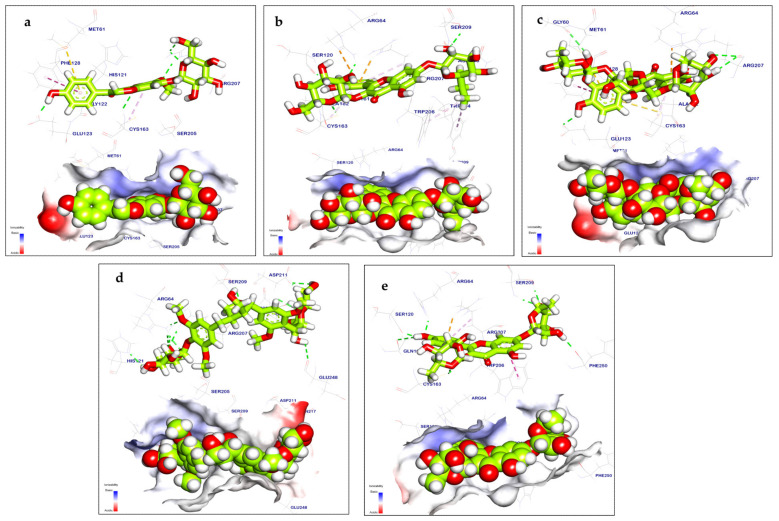
Mapping surfaces and 3D orientation of compounds docked in caspase-3: (**a**) tectoridin; (**b**) quercetin-3,7-*O*-diglucoside; (**c**) manghasin; (**d**) salvadoraside; (**e**) kaempferol 3,7-*O*-alpha-L-dirhamnoside.

Tectoridin displayed an energy binding value of −7.59 kcal/mol^−1^ against caspase-3. It formed hydrogen bonds with Glu123, Gly122, Cys163, and Arg207, as well as one Pi–sulfur interaction with Met61 and one Piinteraction with Phe128 and Cys163 (Figure 12a). Quercetin-3,7-*O*-diglucoside displayed a binding mode with caspase-3, resulting in an energy binding value of −8.62 kcal/mol^−1^ against caspase-3. It generated six hydrogen bond interactions with Ser120, Gln161, Cys163, Arg64, Ser209, and Asn208, as well as three connections between Pi–alkyl Trp214, Cys163, and Trp206; it also showed two Pi–cation interactions with Arg207 and Arg64 (Figure 12b). Manghaslin demonstrated a binding mode with an energy binding value of −8.38 kcal/mol^−1^. It generated four hydrogen bonds with Arg107, Gly60, and Glu123 and two Pi–sulfur interactions with Met61 and Cys161. It also showed two Pi–Pi stacked and Pi–alkyl interactions and one Pi–cation interaction with Phe128, Cys163, and Arg64 (Figure 12c). Salvadoraside showed a binding mode with caspase-3, resulting in an energy binding value of −9.88 kcal/mol^−1^. Salvadoraside formed eleven hydrogen bond interactions with Glu248, Gln217, Asp211, Asn208, Ser209, Arg207, Arg64, and His121 (Figure 12d). Kaempferol-3,7-*O*-*α*-L-dirhamnoside displayed a binding mode with caspase-3, yielding an energy binding value of −8.58 kcal/mol^−1^. Kaempferol-3,7-*O*-*α*-L-dirhamnoside formed seven hydrogen bonds with Cys163, Ser120, Gln161, Arg64, Ser209, and Phe250and additionally showed three Pi interactions with Arg207, Trp20, and Trp206 (Figure 12e). Table 4 shows the free binding energy ∆G (kcal/mol) of the identified metabolites by LC-ESI-QTOF-MS/MS in terms of interactions with the target region ofcaspase-3. PDB ID: 2J31.

### 2.4. Acute Toxicity

#### 2.4.1. Acute Toxicological Evaluation

No signs of physical alteration were found in either the treated or the control animals. There were no indications of loneliness, idleness, or itching. Every animal showed typical behavior; they all ate and drank normally (Table S1); and there were also no signs of diarrhea, drowsiness, tremors, convulsion, excitation, or breathing changes. All animals survived throughout the 14-day experiment period, without showing any symptoms of toxicity or fatalities.

#### 2.4.2. Histopathology

**Figure 13:**
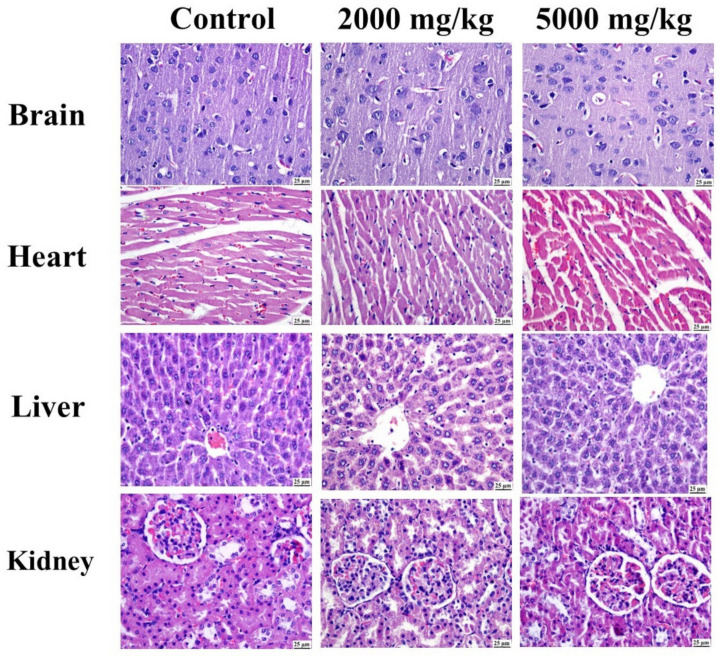
Photomicrographs (H&E) showing normal histology of different organs in control group and apparently normal structure of different organs in both 2000 and 5000 mg/kg treated groups.

As illustrated in Figure 13 and Figure S29, the control group showed a normal histological structure without any detectable alterations. Likewise, both groups treated at different concentrations showed a clearly normal histology in the different organs.

## 3. Discussion

Previously, CEE exhibited a beneficial effect as a cytotoxic agent on HT-29 cells, as indicated by an IC_50_ value of 13.28 μg/mL, with a dose-dependent response [21]. Herein, this potent cytotoxic activity was clarified in terms of arresting the cell cycle, promoting apoptosis, and inhibiting cell migration/metastasis, as well as upregulating the transcription of apoptotic markers (BAX and caspase-3) and downregulating the transcription of both BCL-2 and VEGF. The effect of CEE on the progression of the cell cycle was found in the form of a notable pause in the G2/M stage. Changes that occur to the G2/M checkpoint prevent the cell from entering mitosis, indicating that DNA injury is present [49]. Apoptosis evasion is a prominent feature of cancerous cells, and targeting this pathway is an important therapeutic strategy [50]. In the current study, the potential of CEE to trigger apoptosis was investigated utilizing the FTIC Annexin V/PI method. The results showed that CEE exhibited significant cytotoxic effects, which correlated with the induction of apoptosis. The trigger of programmed cell death in HT-29 cells by CEE can be attributed to the modulation of the mitochondrial apoptosis pathway. The regulation of cellular apoptosis is influenced by antiapoptotic and proapoptotic proteins [51]. The administration of CEE led to the overexpression of caspase-3 and BAX mRNA and the suppression of BCL-2. Cell migration is a crucial aspect of cancer metastasis [52]. CEE treatment demonstrated the significant inhibition of cell migration and metastatic progression in HT-29 cells, indicating its potential as an effective antitumor agent that reduces the occurrence of metastasis. Furthermore, it is known that VEGF and its receptor (VEGFR) are crucial to the development of pathological angiogenesis, as observed in cancer [52]. In the current study, CEE treatment significantly inhibited VEGF mRNA expression in HT-29 cells, suggesting a promising antimetastatic effect.

This potent cytotoxicity probably pertains to its richness in phytoconstituents, especially polyphenolics, having total flavonoid content of 7.16 mg/g of dry extract and high total phenolic acid content (68.18 mg/g dry extract) [21]. Multiple previous reports have mentioned the capabilities of various phytoconstituents in the *Clematis* genus to induce apoptosis in different cancer cells [53]. Many mechanisms have been assumed to explain this potent cytotoxic activity, including the inhibition of cell line migration/invasion in the late-stage breast cancer (MDA-MB-231) cell line [54] and the hindering of cell cycle progression [55]. For instance, boehmenan, a lignan isolated from *C. armandii*, was reported to hinder the progression of the cell cycle within A431 cells [55]; hederagenin saponin derived from *C. ganpiniana* has been found to induce programmed cell death in human breast cancer (MCF-7) and MDA-MB-231 cell lines [56]; and a mannose-binding lectin from *C.montana* has shown dose-dependent apoptosis induction in L929 cells [53].

The current study illustrates the metabolic profiling of CEE using high-resolution LC-ESI-QTOF-MS/MS analysis. Twenty-six metabolites were identified, belonging to phenolic compounds (phenolic acids, flavonoids, lignans, phenylethanoids, and phenylpropanoids), fatty acids, and triterpenoids. Three phenolic compounds have been identified in *C. cirrhosa*, including three hydroxycinnamic acids (caffeic acid, *p*-coumaric acid, and ferulic acid), one phenylethanoid (hydroxytyrosol), and one phenylpropanoid glycoside (calceolarioside B). They have been previously reported in different *Clematis* species. In a previous study by Chohraet al., caffeic acid and ferulic acid were identified in the methanol extract of *C. cirrhosa* using high-performance liquid chromatography [42,46,57]. Two lignans were identified as salvadoraside and pinoresinol, which have been previously found in other *Clematis* species [15,58]. Fifteen previously reported flavonoids in different *Clematis* species have been identified [42,46,57,59,60,61]. Furthermore, *C. cirrhosa* contains triterpenoids such as hederagenin and oleanolic acid, as well as two fatty acids, hydroxy octadecadienoic acid and hydroxy palmitic acid, which have been previously found in other *Clematis* species as well [48].

These results are consistent with earlier studies revealing the cytotoxicity of the identified metabolites, including orientin, which has been found to considerably reduce the growth of liver cancer cells [62]. acacetin, another flavonoid found in *C. cirrhosa*, has been observed to suppress the growth of Jurkat cells by triggering apoptosis [63]. Moreover, caffeic acid induces apoptosis and morphological changes in breast cancer cells [64], and it also exhibits antiproliferative effects and modulates gene-specific DNA methylation in human breast tumor cells [65]. Ferulic acid shows apoptotic activity against ACHN renal carcinoma cells [66], while hydroxy tyrosol exhibits significant antiproliferative and proapoptotic effects on human tumoral cell lines such as HL60 [67]. Additionally, pinoresinol has demonstrated cytotoxicity, antiproliferative effects, and pro-oxidant activity in human breast tumor cells [68]. In addition, studies have found that oleanolic acid can trigger a cell cycle halt at the G2/M phase in HepG2 cells [69]. Similarly, it has been demonstrated that hederagenin boosts the cytotoxicity of paclitaxel and cisplatin in lung cancer cells by inhibiting the flow of autophagy and encouraging the build-up of reactive oxygen species, which in turn encourages apoptosis [70].

In silico molecular docking methods serve a dual purpose: they are highly valuable in identifying potential binding sites and in discovering novel molecules capable of binding to known sites [71]. In the current investigation, the metabolites were subjected to simulated molecular docking investigations after high-resolution LC-ESI-QTOF-MS/MS spectrometry analysis to identify possible target molecules for the cytotoxic and apoptosis-inducing effects. The docking results revealed good binding interactions of calceolarioside B, quercetin-3,7-*O*-diglucoside, manghaslin, quercetin 7-*O*-α-L-rhamnopyranoside, and luteolin, interacting with apoptotic proteins such as VEGFR-2 tyrosine kinase with high binding energy (−6.44 to −9.68Kcal/mol). Furthermore, compounds like tectoridin, quercetin-3,7-*O*-diglucoside, manghaslin, salvadoraside, and kaempferol-3,7-*O*-α-L-dirhamnoside demonstrate interactions with caspase-3 with high binding energy (−7.59 to −9.88 Kcal/mol), suggesting their potential involvement in apoptotic pathways. Our docking experiment suggests that the identified compounds act as suppressors of VEGFR-2 and caspase-3, which aligns with the findings of quantitative reverse transcription polymerase chain reaction (qRT-PCR) analyses, demonstrating the ability to induce apoptosis.

To effectively fulfill worldwide usage requirements, recent research has focused on traditional medicines’ safety and effectiveness [72]. It is essential to evaluate extracts’ toxicity in accordance with accepted standards before the development of phytomedicines derived from medicinal plants [73]. Preclinical dose determination is aided by this procedure, providing information on side effects [74]. There is currently no published research available on the toxicity of CEE. However, a study conducted on *C. terniflora* found that the oral administration of a water extract and 70% ethanol extract at a dosage of 0.1 g/kg body weight did not result in any acute toxicity [75]. The present study evaluated the acute toxicity in male rats. The oral gavage of CEE at up to 5000 mg/kg did not cause any mortality or significant changes in clinical signs or organ histopathology in male rats. The LD50 value of CEE was found to be greater than 5000 mg/kg body weight, which indicates that it can be considered as having low toxicity [76].

## 4. Materials and Methods

### 4.1. Plant Material

*C. cirrhosa* L. was harvested during the flowering season (April 2021) from the Ajloun Highlands (32.332687, 35.751785) in Jordan. Mr. Sameh Khatatbeh, an expert in flora study and an ecological researcher at the Royal Society for the Conservation of Nature (Amman, Jordan), verified the taxonomic identification of the plant material. A sample was kept at the Department of Pharmacognosy, Faculty of Pharmacy, Cairo University, Egypt (voucher number 20-06-2021 Ι). The plant aerial parts were gathered, air-dried in the shade, ground into a fine powder (mesh size: 0.2–0.636 mm), and kept in firmly closed glass bottles until use. The powders were macerated in 70% ethanol in three separate extractions with constant shaking. The obtained extracts were evaporated under reduced pressure using a rotary evaporator (Stuart, Staffordshire, UK) until complete dryness [21].

### 4.2. Materials

Ethanol was obtained from Alpha Chemika, India; SRB was provided by Sigma Aldrich (Steinheim, Germany); and DOX was supplied by Pfizer, Bentley, Austria. The following equipment was used: iScript one-step reverse transcription polymerase chain reaction (qRT-PCR) with SYBR Green kit (Bio-Rad Laboratories, Hercules, CA, USA), catalog # 170-8893; propidium iodide flow cytometry kit (Abcam, Shanghai, China), catalog # ab139418; Annexin V–FITC apoptosis detection kit (BioVision, Milpitas, CA, USA), catalog # K 101-25; RNeasy mini kit (Qiagen, Germantown, MD, USA), catalog # 74536. Acetonitrile, 0.1% formic acid (*v*/*v*) in water, and sodium hydroxide were procured from Thermo Fisher Scientific (Pittsburgh, PA, USA). Methanol was obtained from Alfa Chemistry (Holbrook, NY, USA). Milli-Q water was purchased from Merck (Darmstadt, Germany), while ammonium formate was acquired from Spectrum (Los Angeles, CA, USA). Cell lines were provided by Nawah Scientific, Inc. (Mokatam, Cairo, Egypt).

### 4.3. Cytotoxicity Evaluation 

In a previous study, we revealed that CEE exhibits cytotoxic properties against HT-29colorectal cancer cells [21]. For further analysis, HT-29 cells were grown in 75 cm^2^ bottles at a density of 1 × 10^6^ cells/mL in Dulbecco’s Modified Eagle Medium enriched with 10% fetal bovine serum and with antibiotics added.The cells were left to incubate for 72h with CEE (13.28 μg/mL) or DOX (0.48 μg/mL) at concentrations determined by their respective IC_50_ values obtained from the SRB assay. Negative controls consisted of cells without any treatment. Cell extraction was performed using trypsin–EDTA [77].

### 4.4. Cell Cycle Analysis

Flow cytometry was employed to evaluate the phase distribution of HT-29 cells following treatment with CEE or DOX. The HT-29 colorectal cancer cells were trypsinized and then kept overnight at 4 °C in 66% ethanol. Two washes with phosphate-buffered saline (PBS) were performed, and the fixed cells were resuspended in PBS containing 0.1 mg/mL RNase and 50 µg/mL propidium iodide (Beyotime, Shanghai, China). The cells were then incubated at 37 °C for 30 min in the absence of light. Ultimately, an LSR II FACS flow cytometer (BD Biosciences, Franklin Lakes, NJ, USA) was used to examine the cells [78].

### 4.5. Flow Cytometry-Based Annexin V–FITC/PI Assay for Detection of Apoptosis

To further investigate apoptosis, an Annexin V–FITC apoptosis detection kit was utilized. After being extracted by centrifugation, the cells were again suspended in 1X binding buffer. Then, 5 μL of PI (50 mg/mL) and 5μLof Annexin V–FITC were added to the cell suspension. Following a 5min incubation period, the flow cytometry technique was employed to analyze the Annexin V–FITC binding. Fluorescence measurements were taken using an FITC signal detector [78].

### 4.6. Scratch Wound Healing 

The assay for wound healing was employed to appraise the potential inhibitory impacts on the migration and metastasis of cells. After being planted at a density of 1 × 10^5^ cells/mL in 24-well plates, the HT-29 cells were incubated until they reached confluence. Once the cells formed a complete monolayer, a scratch or gap was created using a 1 mL pipette tip, simulating a wound. The cells were then washed with 500 μL of PBS after the culture medium was withdrawn. Subsequently, 500 μL of full culture medium with either DOX or CEE was added. Immediately after replacing the medium, images were captured at T = 0 and again at 48 h using an inverted microscope (Leica DFC290, Wetzlar, Germany). The closure of the scratch area was quantified by measuring the average percentage of closure of the gap. These experiments were performed in triplicate [79,80,81,82].

### 4.7. mRNA Transcription Levels of VEGF, BAX, BCL-2, and Caspase-3

Total RNA was extracted using the RNeasy mini kit, following the manufacturer’s instructions. For the qRT-PCR, gene-specific primers (listed in Table 5) were employed in combination with the SYBR Green PCR Master Mix and Revert Aid Reverse Transcriptase. The cycle threshold (CT) value of every individual sample was compared to that of the positive control group utilizing the ratio 2^−ΔΔct^, and the relative expression was reported as a fold change. To normalize the data, glyceraldehyde-3-phosphate dehydrogenase (GAPDH) was employed as the housekeeping gene [83,84]. The Rotor-Gene 6000 Series Software 1.7 was used to analyze the genes.

### 4.8. LC-ESI-QTOF-MS/MS Analysis

#### 4.8.1. Sample Preparation and Injection

To prepare a stock solution, 50 mg of dried extract was first diluted with 1000 μL of a reconstituted solvent mixture (water, methanol, and acetonitrile, 2:1:1, *v*/*v*) using a vortex for two minutes, followed by ultrasonication for ten minutes at 30 kHz, to reach a final concentration of 2.5 μg/μL. In both negative and positive modes, a volume of ten μL from the final concentration solution was introduced into the LC-ESI-QTOF-MS/MS system. The reconstituted solvent was administered as a blank in ten μL increments [85]

#### 4.8.2. LC-ESI-QTOF-MS/MS Conditions

The analysis was conducted utilizing a Shimadzu Exion LC system (Kyoto, Japan), combined with the X500 QTOF system (AB Sciex, Framingham, MA, USA). Chromatographic separation was performed using a Poroshell 120 EC-C18 column (2.1 × 100 mm, 3 μm) (GL-Science, Torrance, CA, USA). The column’s temperature was maintained at 50 °C. The mobile phase was composed of0.1% formic acid in water (A) and acetonitrile (B). The gradient elution protocol was customized to optimize the separation of metabolites, with a gradual increase in the proportion of solvent B from 5% (0 to 5 min) to 95% over a period of 25 to 30 min. The column was then brought back to its initial conditions with a decrease in the proportion of solvent B to 5% over the next 8 min. After holding at 95% B for 2 min, the column was maintained at 5% B for 8 min before ending the run. A constant injection volume of 5 μL was employed, and the mobile phase flow rate had to be set at 0.3 mL/min. Mass spectrometry analysis was performed using the X500 QTOF instrument, which was outfitted with a turboion spray source operating in electrospray ionization (ESI) mode. Full-scan SWATH screening was used. Mass spectrometry was run in both positive and negative ESI modes, with the following settings: capillary voltage, 4000 V (negative) and 5000 V (positive); CAD gas, 7psi; pressure of nebulizer, 50 psi; drying gas temperature, 10 L/min; sheath gas temperature, 500 °C; collision energy, 35 ± 15 eV. The mass spectra were recorded by scanning the mass ranging from *m*/*z* 20 to 1200 in MS mode [78].

#### 4.8.3. Data Processing

Metabolite extraction and identification were conducted utilizing a variety of tools. The raw MS files were converted using MS Convert (a free and open-source program) into forms that could be processed by Mzmine 2.53 tools. For peak detection, and feature detection, metabolite identification using the MS data, Mzmine 2.53 (an open-source proteomics program) was utilized. Using algorithms for isotope pattern recognition and wavelet modification, Mzmine found peaks and fragments in the mass spectra. Based on their retention times, the identified features were sorted into chromatograms. To find known metabolites, Mzmine compared the identified attributes against metabolite databases such as HMDB, KEGG, PubChem Compound, etc. The provisional identification of the compounds was further verified by comparing them with the reference literature.

### 4.9. Molecular Docking Study

The AutoDock Vina V.1.5.7 software was utilized to perform the ligand–protein docking of the investigated metabolites against VEGFR-2 TK and caspase-3. For the binding modes, active site residues were created using ligands obtained from the Research Collaboratory for Structural Bioinformatics (RCSB) Protein Data Bank (codes: 4ASD and 2J31). Prior to docking, the targeted proteins underwent preparation steps, including the removal of water molecules, addition of missing amino acids, correction of unfilled valence atoms, and CHARMM force field was used to minimize the energy of protein peptides. The proteins’ essential amino acids were chosen and prepared for evaluation. The 2D structures of the compounds under investigation were created in Chem-Bio Draw Ultra 17.0 and saved in SDF file format. The ligands underwent protonation and energy minimization using the MMFF94 force field with a root-mean-square deviation (RMSD) of 0.1 kcal/mol. Ligands were protonated and energy-minimized. The structures that underwent energy minimization were utilized for the subsequent docking process. The docking algorithms were employed with the target pocket held rigid, while allowing flexibility for the ligands. Throughout refinement, every molecular structure was enabled to create twenty different interacting poses with the protein. The docking scores, corresponding to the binding affinity, were documented for the best-fitting poses of the tested compounds within the active site of VEGFR-2 TK. The 3D orientation of the ligands with the protein was created using the Discovery Studio 2016 visualizer software [85].

### 4.10. Acute Toxicity Study

This study adhered to the guidelines provided by the Organization of Economic Cooperation and Development (OECD) (Paris, France) for the acute toxicity class method (Guideline No. 423) and was approved by the Research Ethics Committee at the Faculty of Pharmacy, Cairo University, Egypt, with the ethical approval number of MP (2820).

#### 4.10.1. Animals

Fifteen adult male Wistar albino rats (170–220 g) were supplied by the National Research Centre, Doki, Egypt and kept in polypropylene cages at the animal rearing facility at the Faculty of Pharmacy, Cairo University, Cairo, Egypt, under an appropriate laboratory environment with suitable humidity (40–75%), temperature (23 ± 2 °C), and light (12 light–dark cycle) levels, with ample access to food and water. All rats were acclimatized to the new environment for 14 days before the study. At the start of the experiment, the rats were randomly allocated into three groups (5 animals per group) with the assistance of the lab technician and then marked and weighed. The control group received a vehicle (distilled water), while the treated groups received, by oral gavage, either a single dose of 2000 or 5000 mg/kg body weight of the freshly prepared CEE. After dosing, the general characteristics of the animals and clinical signs of toxicity were observed during the first 30 min and then after 2, 4 and 6 h and once daily over 14 days. At the end of the study, the animals were euthanized under deep anesthesia, and their organs were isolated and kept in formalin for histological examination [86].

#### 4.10.2. Histopathological Examination

The brain, heart, liver, and kidney fragments were preserved in 10% buffered formalin (pH 7.4). Subsequently, the fragments were dehydrated in absolute ethyl alcohol, immersed in xylene, embedded in paraffin, sectioned, and stained with hematoxylin–eosin (H&E). Finally, all tissue sections were examined under a microscope.

## 5. Conclusions

This study illustrates, for the first time, the diversity in the chemical profiling of the *C. cirrhosa* ethanol extract using LC-ESI-QTOF-MS/MS. Twenty-six metabolites were identified, belonging to phenolic compounds (phenolic acids, flavonoids, lignans, phenylethanoids, and phenylpropanoids), fatty acids, and triterpenoids. On HT-29 cells, the extract exhibited strong antiproliferative and proapoptotic properties and inhibited cell migration. Moreover, the results of the docking experiments suggested that some of these compounds could inhibit VEGFR-2 and caspase-3, supporting the observed effects. Additionally, the acute toxicity study documented the extract’s safety. Finally, such findings highlight the potential of *Clematis cirrhosa* as a valuable candidate for further research in antitumor therapy.

## Figures and Tables

**Table 1:** Metabolites identified in *Clematis cirrhosa* crude extract using LC-ESI-QTOF-MS/MS in the negative mode.

Peak No.	Rt (m)	Mol. Ion (*m*/*z*) [M-H]^−^	Molecular Formula	Error	Identified Compound	Class	MS^2^ Fragments	Ref.
1	0.80	461.1058/-	C_22_H_22_O_11_	−5.61	Chrysoeriol-*O*-hexoside	Methoxyflavon-*O*-glycoside	446.1667, 299.0528, 284.1156	[22]
2	8.31	153/-	C_8_H_10_O_3_	−1.76	Hydroxytyrosol	Phenylethanoid	123.0452	[23]
3	8.49	477.1614/-	C_23_H_26_O_11_	2.75	Calceolarioside B	Phenylethanoid glycoside	315.1071, 179.035, 161.9485	[24]
5	9.23	289.0713/-	C_15_H_14_O_6_	0.3	* Catechin	Flavanol	245.0823, 205.0511	[25]
6	9.25	163.0402/-	C_9_H_8_O_3_	4.18	*p*-Coumaric acid	Hydroxycinnamic acid	119.9177	[26]
7	9.87	625.1382/-	C_27_H_30_O_17_	−3.64	Quercetin-3,7-*O*-dihexoside	Flavonol-*O*-glycoside	463.1413, 301.1013	[27]
8	10.2	755.2126/-	C_33_H_40_O_20_	−1.15	Manghaslin	Flavonol-*O*-glycoside	591.1035, 301.0366	[28]
9	10.47	743.2789/-	C_34_H_48_O_18_	3.58	Salvadoraside	Phenylpropanoid lignan	581.1535, 419.1818, 389.1821	[29]
10	10.59	357.1338/-	C_20_H_22_O_6_	−0.04	Pinoresinol	Tetrahydrofuran lignan	151.1139, 136.1222	[30]
11	10.74	447.0912/-	C_21_H_20_O_11_	−3.44	Quercetin-*O*-rhamnopyranoside (Vincetoxicoside B)	Flavonol-*O*-glycoside	301.1383	[31]
12	10.74	447.098/-	C_21_H_20_O_11_	−3.21	Orientin	Flavone-*C*-glycoside	357.0708, 327.0097, 285.2595	[32]
13	11.14	577.1563/-	C_27_H_30_O_14_	2.72	Kaempferol-3,7-*O*-dirhamnoside	Flavonol-*O*-glycoside	431.0993, 285.1348	[33]
14	11.44	463.0869/-	C_21_H_20_O_12_	−1.62	Isoquercitrin	Flavonol-*O*-glycoside	301.1385, 151.0776	[27]
15	11.61	593.150/-	C_27_H_30_O_15_	−1.09	Kaempferol-*O*-rutinoside	Flavonol-*O*-glycoside	285.1159	[34]
16	12.16	193.0496/-	C_10_H_10_O_4_	−2.51	* Ferulic acid	Hydroxycinnamic acid	134.0364	[35]
18	13.34	269.0454/-	C_15_H_10_O_5_	1.49	Genistein	Isoflavone	241.081, 225.9265, 197.9058, 143	[36]
19	13.45	301.0355/-	C_15_H_10_O_7_	0.9	Quercetin	Flavonol	273.1587, 179.0358, 151.9203, 107.0858	[37]
20	13.5	285.0417/-	C_15_H_10_O_6_	6.27	Luteolin	Flavone	241.0697, 175.9944, 151.0242, 133.1026	[38]
22	19.08	295.2266/-	C_18_H_32_O_3_	−2.44	Hydroxy octadecadienoic acid	Fatty acid	277.2170, 171.8337	[39]
23	19.87	471.348/-	C_30_H_48_O_4_	1.2	Hederagenin	Pentacyclic oleanane-type triterpene	405.2010, 393.1453	[40]
25	21.8	271.2274/-	C_16_H_32_O_3_	0.3	Hydroxy palmitic acid	Fatty acid	253.2179, 225.2179	[39]
26	22.1	455.3531/-	C_30_H_48_O_3_	1.27	Oleanolic acid	Pentacyclic oleanane-type triterpene	407.1748	[41]

* Previously identified in *Clematis cirrhosa* [42].

**Table 2:** Metabolites identified in *Clematis cirrhosa* crude extract using LC-ESI-QTOF-MS/MS in the positive mode.

Peak No.	Rt (m)	Mol. Ion (*m*/*z*) [M+H]^+^	Molecular Formula	Error	Identified Compound	Class	MS^2^ Fragments	Ref.
4	8.83	-/181.0505	C_9_H_8_O_4_	2.85	* Caffeic acid	Hydroxycinnamic acid	163.0386	[43]
17	13.25	-/417.121	C_21_H_20_O_9_	5.86	Puerarin	Isoflavone glycoside	399.8033, 297.1073, 351.2390, 267.1618	[40]
21	18.33	-/463.133	C_22_H_22_O_11_	8.96	Tectoridin	Isoflavone glycoside	301.1407, 286.2915	[44]
24	20.04	-/285.0736	C_16_H_12_O_5_	7.02	Acacetin	Flavone	270.2440, 242.0699, 153.1299, 133.0979	[45]

* Previously identified in *Clematis cirrhosa* [46].

**Table 3:** Free binding energy (kcal/mol) of identified metabolites by LC-ESI-QTOF-MS/MS in terms of interactions with the target region of VEGFR-2 TK. PDB ID: 4ASD.

Ligand	RMSD Value (Å)	Docking Score (kcal/mol)	Interactions	
			Hydrogen Bonds	PiInteractions
Calceolarioside B	1.48	−9.20	9	9
Quercetin-3,7-*O*-diglucoside	1.57	−6.44	8	3
Manghaslin	1.92	−9.68	8	9
Quercetin 7-*O*-alpha-L-rhamnopyranoside	1.04	−8.37	6	9
Luteolin	1.33	−7.18	6	8
Sorafenib	1.36	−8.50	5	19

**Table 4:** Free binding energy ∆G (kcal/mol) of identified metabolites by LC-ESI-QTOF-MS/MS in terms of interactions with the target region ofcaspase-3. PDB ID: 2J31.

Ligand	RMSD Value (Å)	Docking Score (kcal/mol)	Interactions	
			Hydrogen Bonds	PiInteractions
Tectoridin	1.75	−7.59	6	3
Quercetin-3,7-*O*-diglucoside	1.47	−8.62	6	5
Manghaslin	1.78	−8.38	4	5
Salvadoraside	1.73	−9.88	11	0
Kaempferol-3,7-*O*-alpha-L-dirhamnoside	1.90	−8.58	7	4

**Table 5:** Primer sequences used for real-time qRT-PCR.

Gene	Forward (5′→3′)	Reverse (5′→3′)
VEGF	5′-TTGCCTTGCTGCTCTACCTCCA-3′	5′-GATGGCAGTAGCTGCGCTGATA-3′
BAX	5′-TCAGGATGCGTCCACCAAGAAG-3′	5′-TGTGTCCACGGCGGCAATCATC-3′
BCL-2	5′-ATCGCCCTGTGGATGACTGAGT-3′	5′-GCCAGGAGAAATCAAACAGAGGC-3′
Caspase-3	5′-GGAAGCGAATCAATGGACTCTGG-3′	5′-GCATCGACATCTGTACCAGACC-3′
GAPDH	5′-GTCTCCTCTGACTTCAACAGCG-3′	5′-ACCACCCTGTTGCTGTAGCCAA-3′

## Data Availability

Data are contained within the article and Supplementary Materials.

## Supplementary Materials

The following supporting information can be downloaded at: https://www.mdpi.com/article/10.3390/ph17101347/s1, Figure S1: Total ion chromatogram of *C. cirrhosa* ethanol extract in the positive ionization mode; Figure S2: Total ion chromatogram of *C. cirrhosa* ethanol extract in the negative ionization mode; Figure S3: MS/MS spectrum of chrysoeriol-*O*-hexoside (Peak 1); Figure S4: MS/MS spectrum of hydroxytyrosol (Peak 2); Figure S5: MS/MS spectrum of calceolarioside B (Peak 3); Figure S6: MS/MS spectrum of caffeic acid (Peak 4); Figure S7: MS/MS spectrum of catechin (Peak 5); Figure S8: MS/MS spectrum of *p*-coumaric acid (Peak 6); Figure S9: MS/MS spectrum of quercetin-3,7-*O*-dihexoside (Peak 7); Figure S10: MS/MS spectrum of manghaslin (Peak 8); Figure S11: MS/MS spectrum of salvadoraside (Peak 9); Figure S12: MS/MS spectrum of pinoresinol (Peak 11); Figure S13: MS/MS spectrum of vincetoxicoside B (Peak 11); Figure S14: MS/MS spectrum of orientin (Peak 12); Figure S15: MS/MS spectrum of kaempferol-3,7-*O*-dirhamnoside (Peak 13); Figure S16: MS/MS spectrum of isoquercitrin (Peak 14); Figure S17: MS/MS spectrum of kaempferol-*O*-rutinoside (Peak 15); Figure S18: MS/MS spectrum of ferulic acid (Peak 16); Figure S19: MS/MS spectrum of puerarin(Peak 17); Figure S20: MS/MS spectrum of genistein (Peak 18); Figure S21: MS/MS spectrum of quercetin (Peak 19); Figure S22: MS/MS spectrum of luteolin (Peak 20); Figure S23: MS/MS spectrum of tectoridin (Peak 21); Figure S24: MS/MS spectrum of hydroxy octadecadienoic acid (Peak 22); Figure S25: MS/MS spectrum of hederagenin (Peak 23); Figure S26: MS/MS spectrum of acacetin (Peak 24); Figure S27: MS/MS spectrum of hydroxy palmitic acid (Peak 25); Figure S28: MS/MS spectrum of oleanolic acid (Peak 26); Table S1: Effect of CEE on body wight changes; Figure S29: Microscopic examination of different brain regions, heart, liver, and kidney.
