# Supplementary material for: Insights into Clematis cirrhosa L. Ethanol Extract: Cytotoxic Effects, LC-ESI-QTOF-MS/MS Chemical Profiling, Molecular Docking, and Acute Toxicity Study
Source: Pharmaceuticals (Basel). 2024 Oct 9;17(10):1347. doi: 10.3390/ph17101347 (PMC11510288; doi:10.3390/ph17101347)
Supplement: Supplementary file 1 [file pharmaceuticals-17-01347-s001.zip › pharmaceuticals-3207956-supplementary.pdf]

SUPPLEMENTARY MATERIAL

Insights into Clematis cirrhosa L. Ethanol Extract: Cytotoxic Effects, LC-ESI-QTOF-MS/MS Chemical Profiling, Molecular Docking, and Acute Toxicity Study

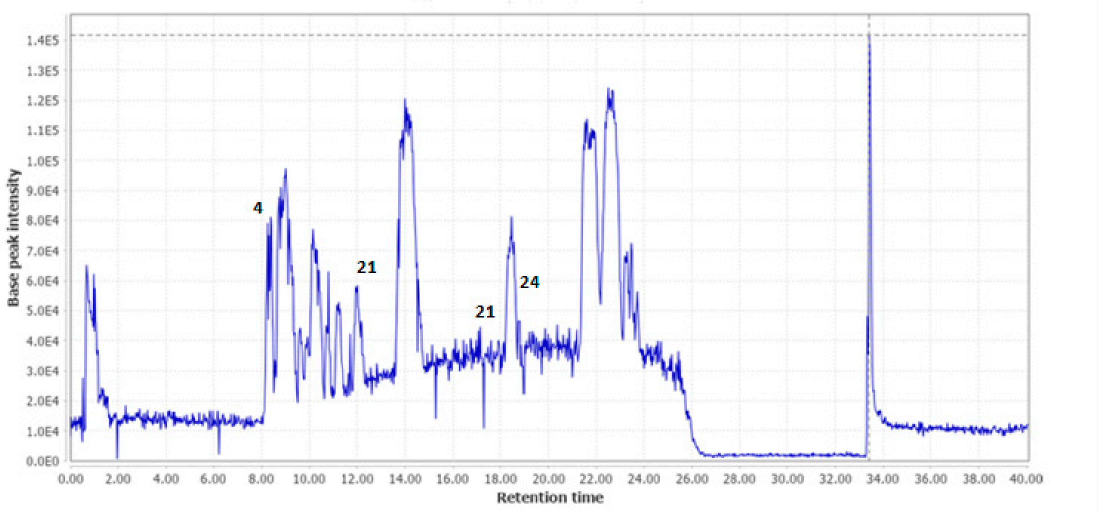

Figure S1: Total ion chromatogram of *C. cirrhosa* ethanol extract in the positive ionization mode

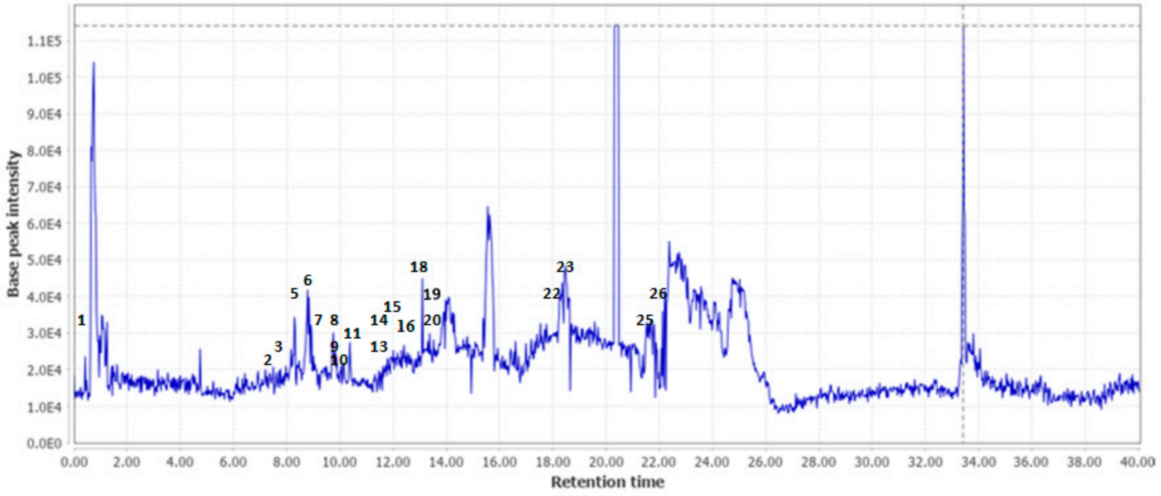

Figure S2: Total ion chromatogram of *C. cirrhosa* ethanol extract in the negative ionization mode

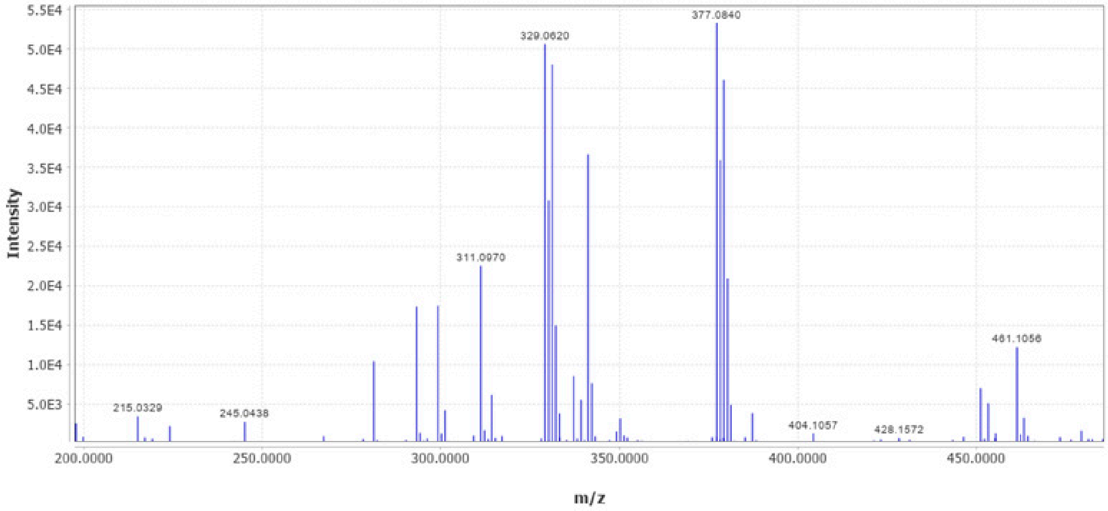

Figure S3: MS/MS spectrum of Chrysoeriol-*O*-hexoside (Peak 1)

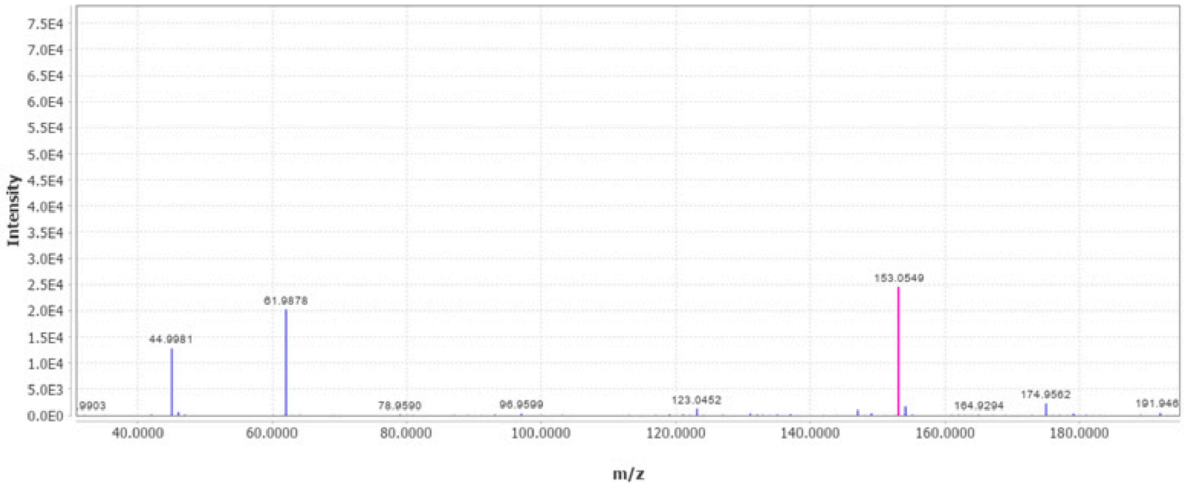

Figure S4: MS/MS spectrum of hydroxytyrosol (Peak 2)

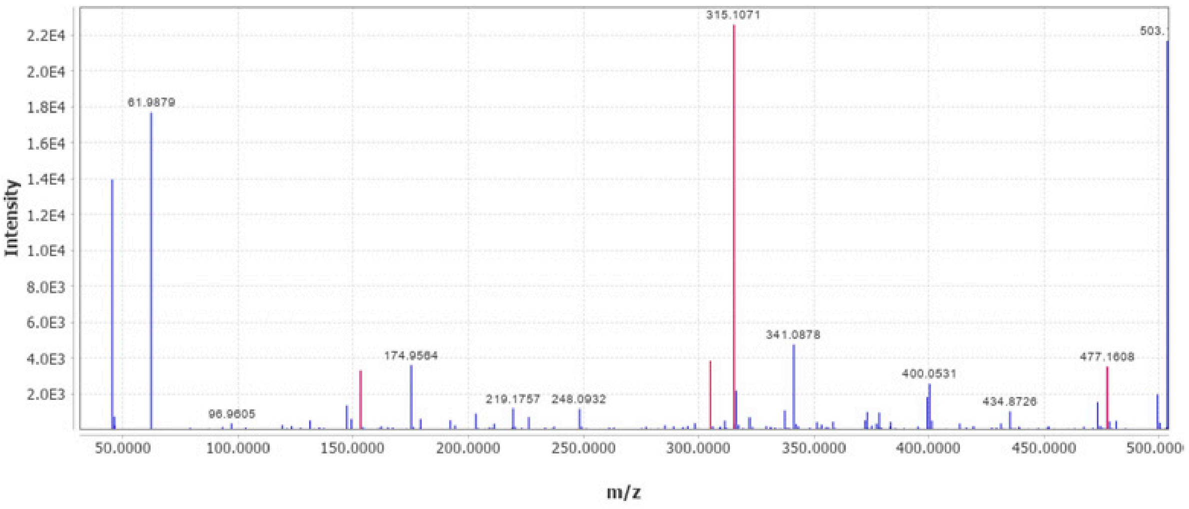

Figure S5: MS/MS spectrum of calceolarioside B (Peak 3)

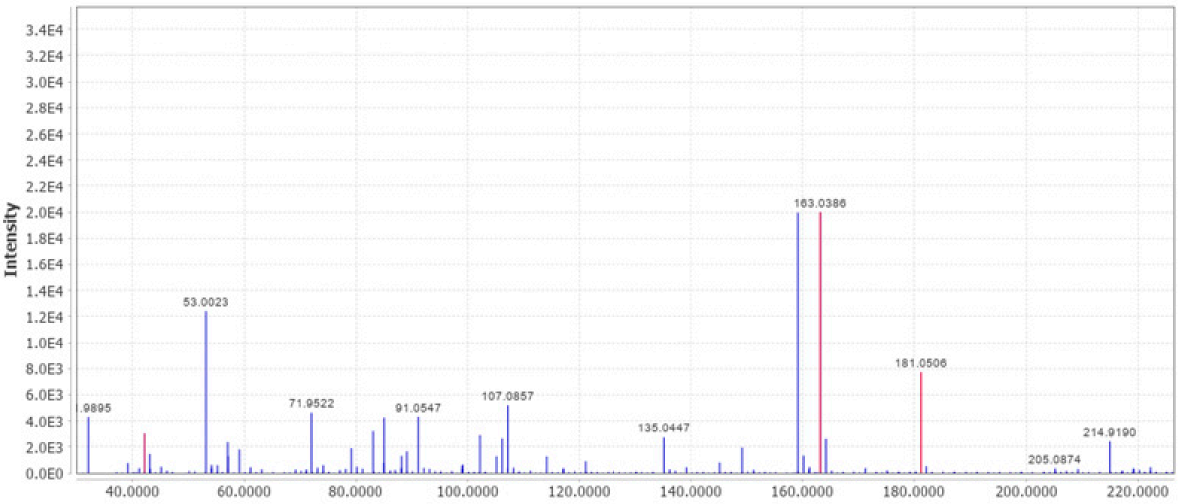

Figure S6: MS/MS spectrum of caffeic acid (Peak 4)

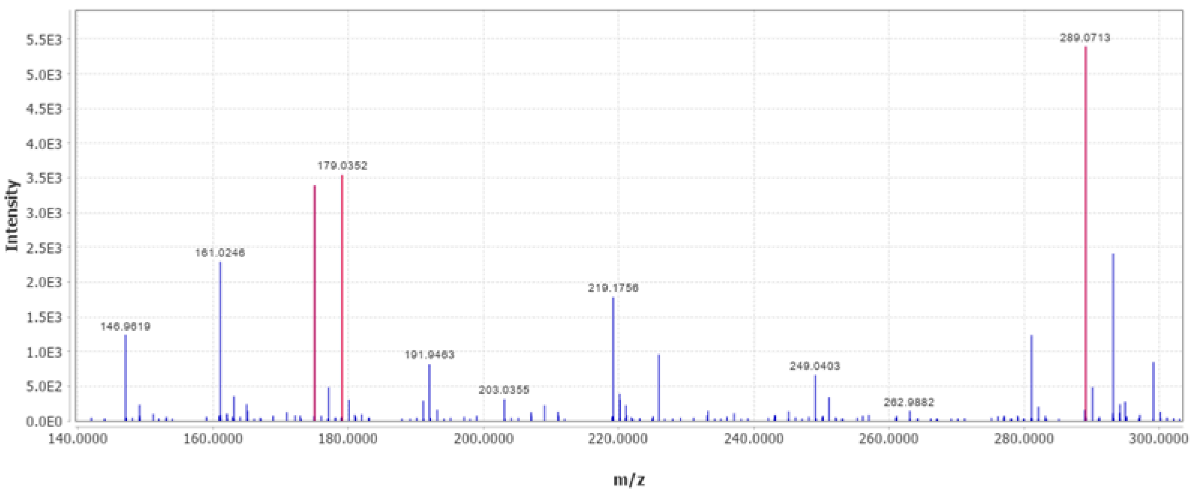

Figure S7: MS/MS spectrum of catechin (Peak 5)

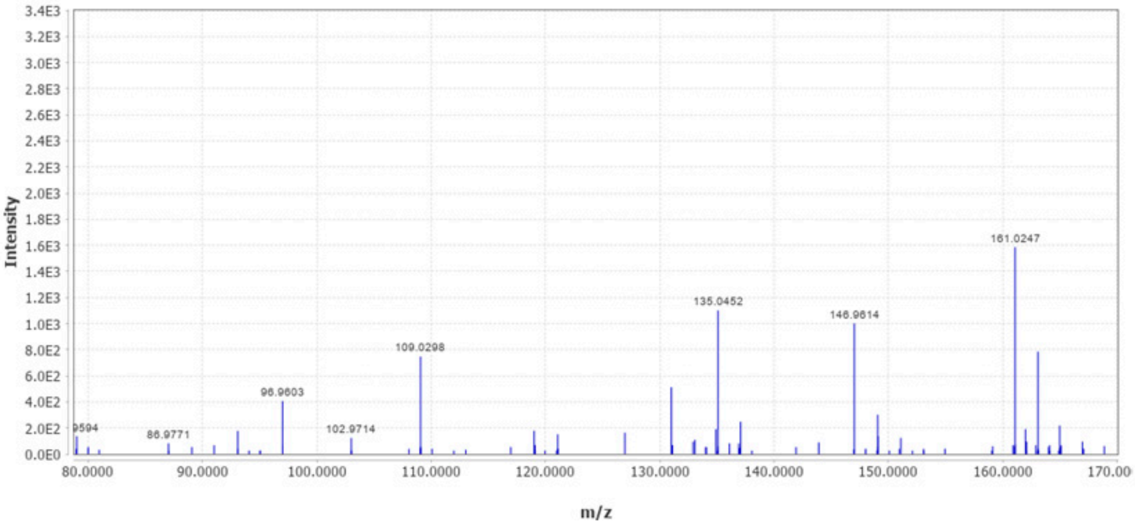

Figure S8: MS/MS spectrum of *p*-coumaric acid (Peak 6)

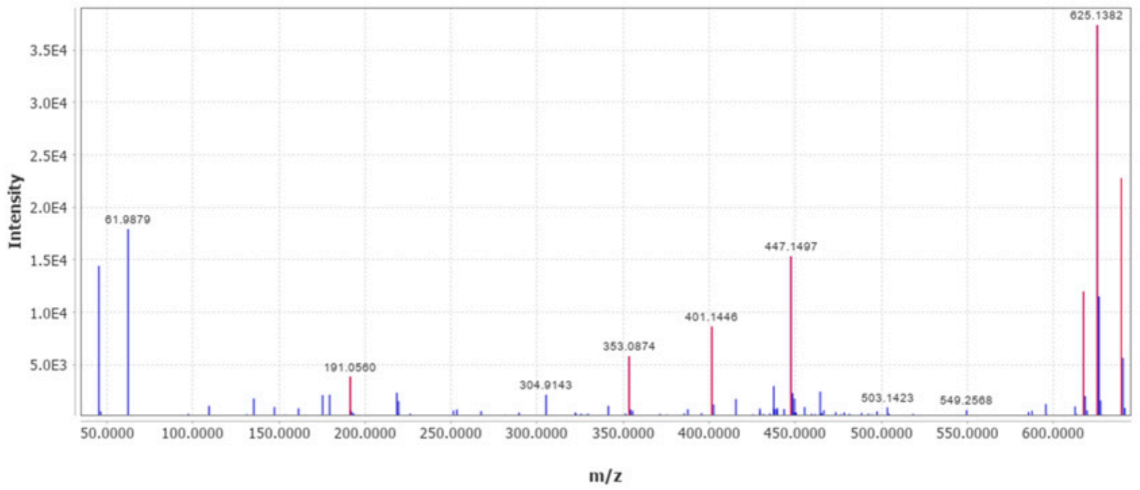

Figure S9: MS/MS spectrum of Quercetin-3,7-*O*-dihexoside (Peak 7)

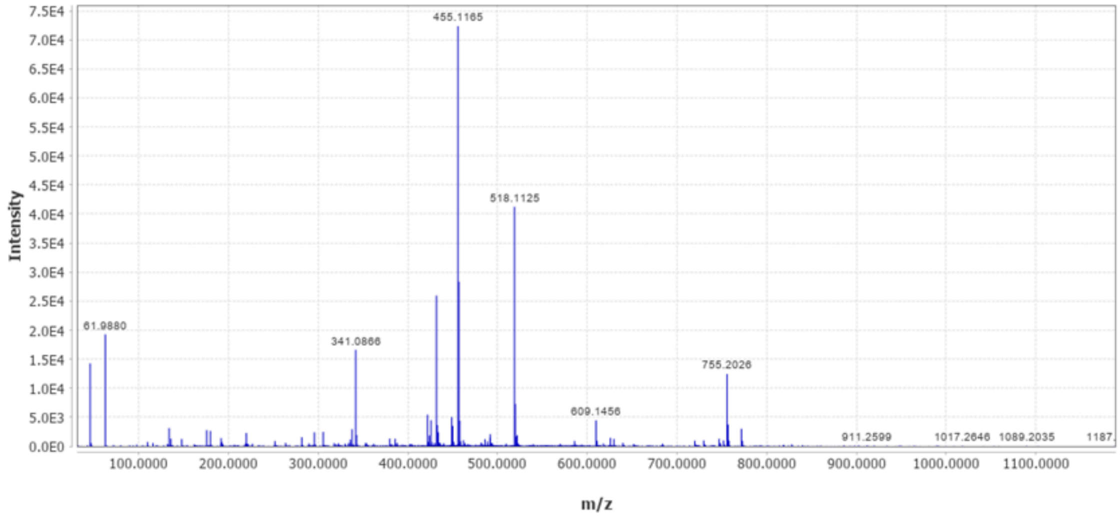

Figure S10: MS/MS spectrum of Manghaslin (Peak 8)

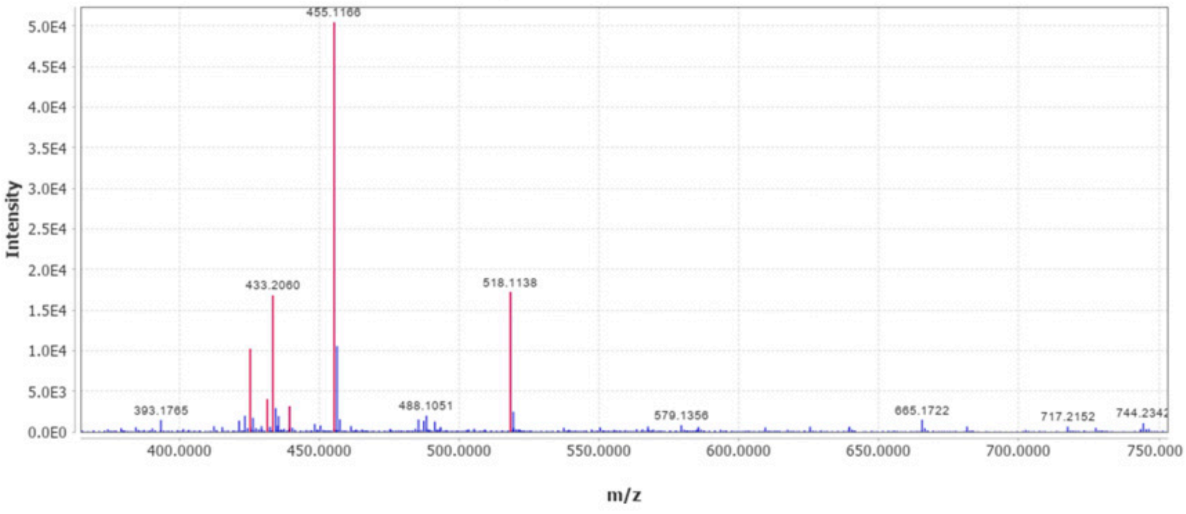

Figure S11: MS/MS spectrum of salvadoraside (Peak 9)

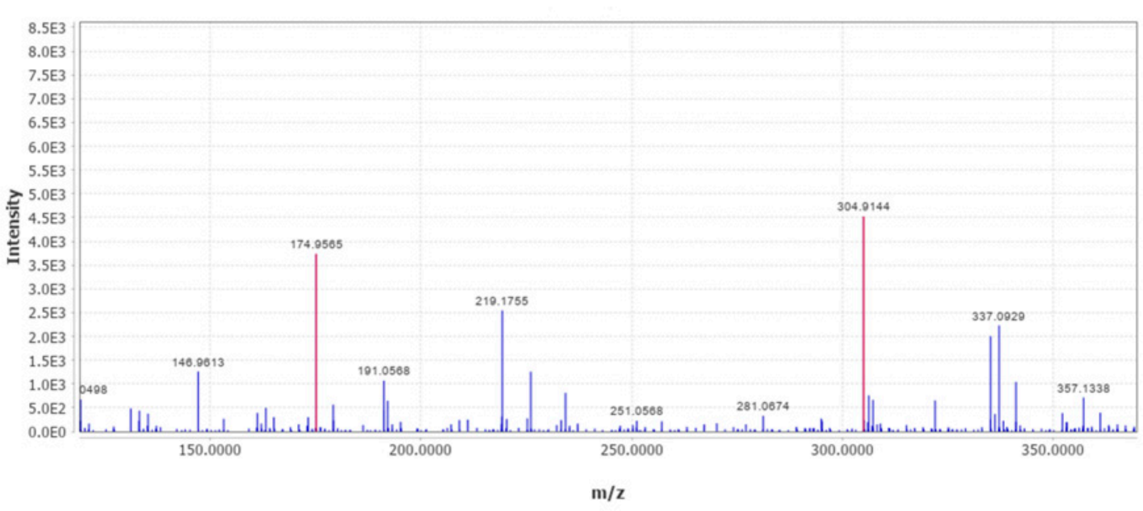

Figure S12: MS/MS spectrum of Pinoresinol (Peak 10)

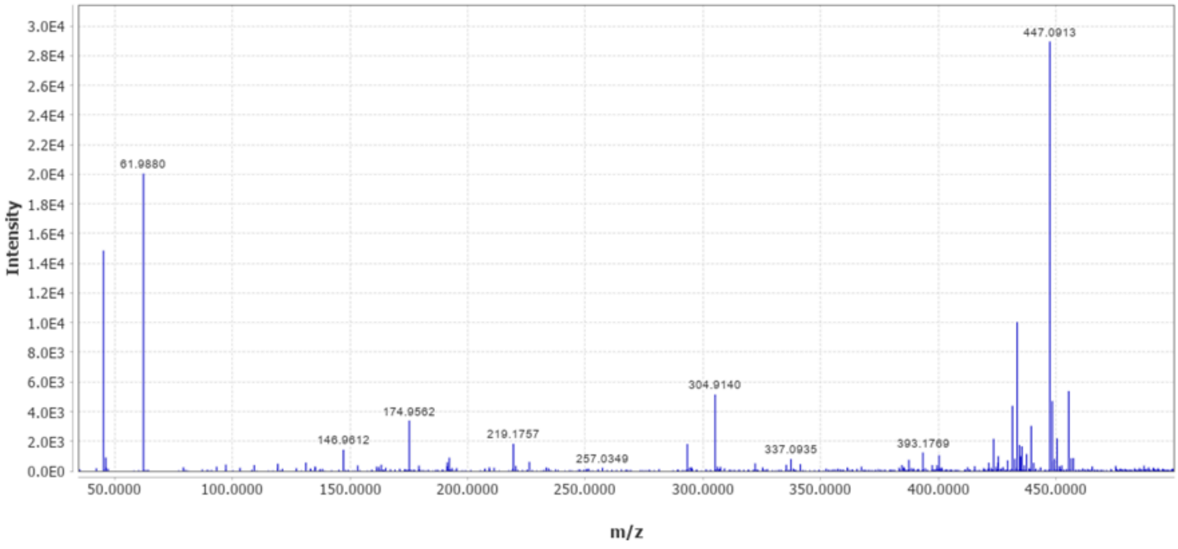

Figure S13: MS/MS spectrum of Vincetoxicoside B (Peak 11)

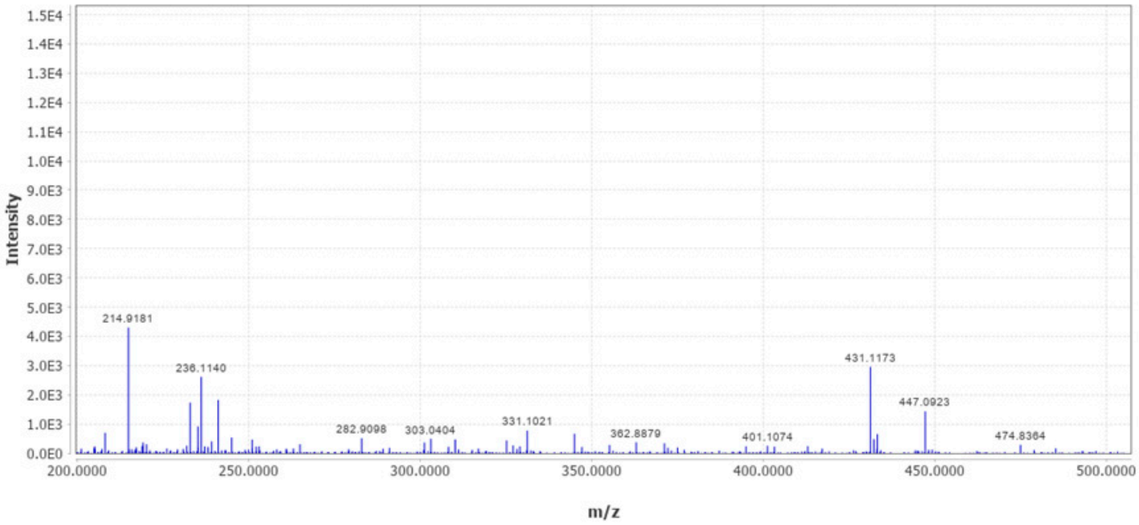

Figure S14: MS/MS spectrum of Orientin (Peak 12)

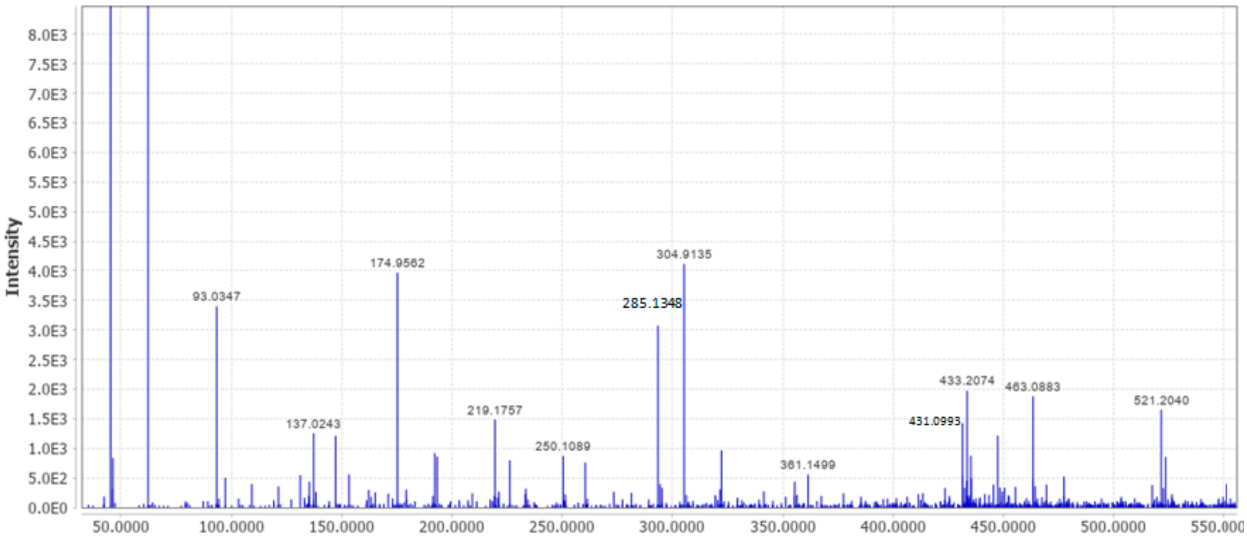

Figure S15: MS/MS spectrum of Kaempferol-3,7-*O*-dirhamnoside (Peak 13)

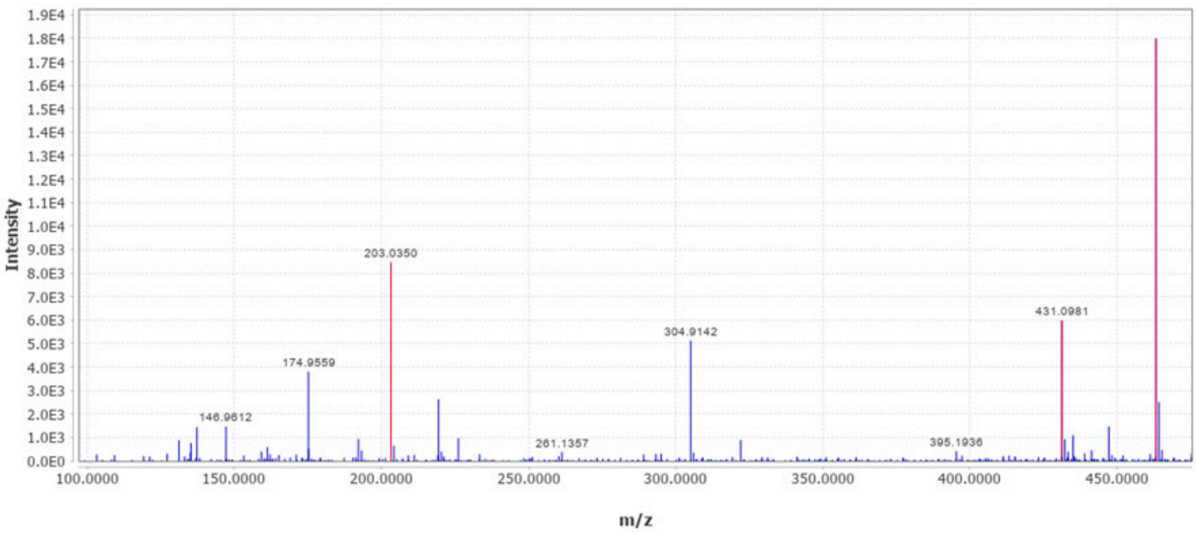

Figure S16: MS/MS spectrum of Isoquercitrin (Peak 14)

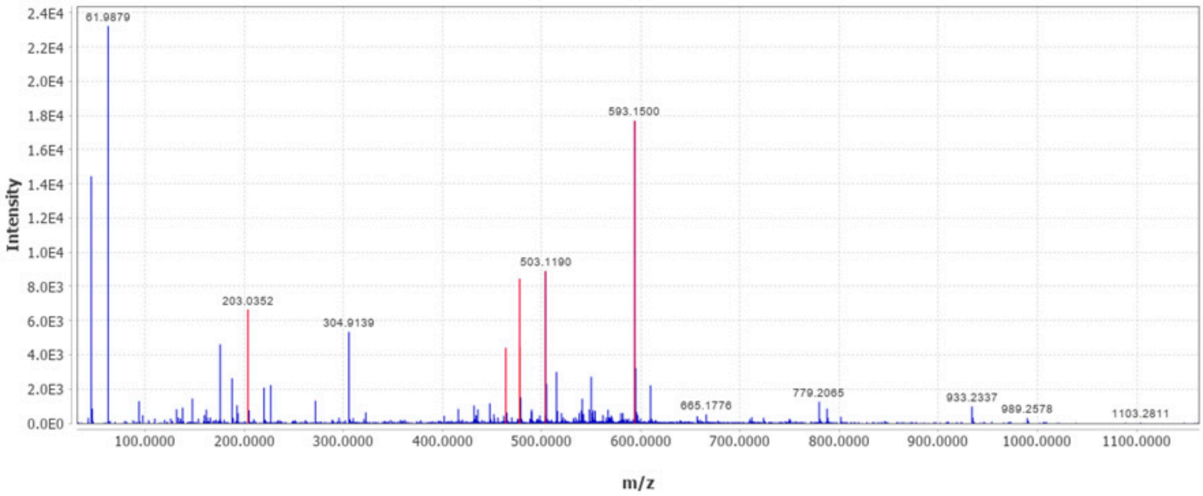

Figure S17: MS/MS spectrum of Kaempferol-*O*-rutinoside (Peak 15)

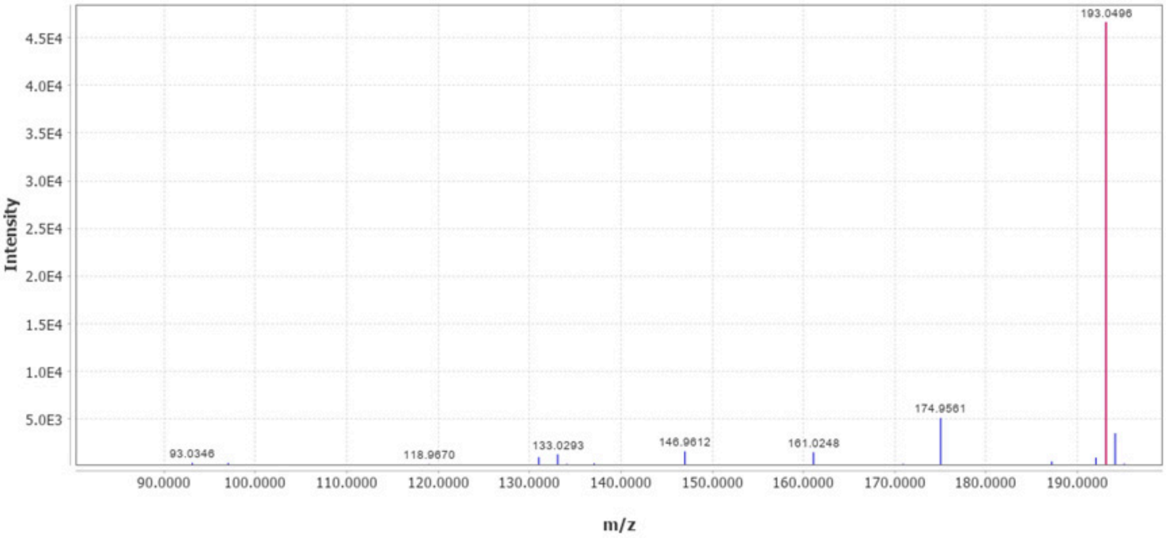

Figure S18: MS/MS spectrum of Ferulic acid (Peak 16)

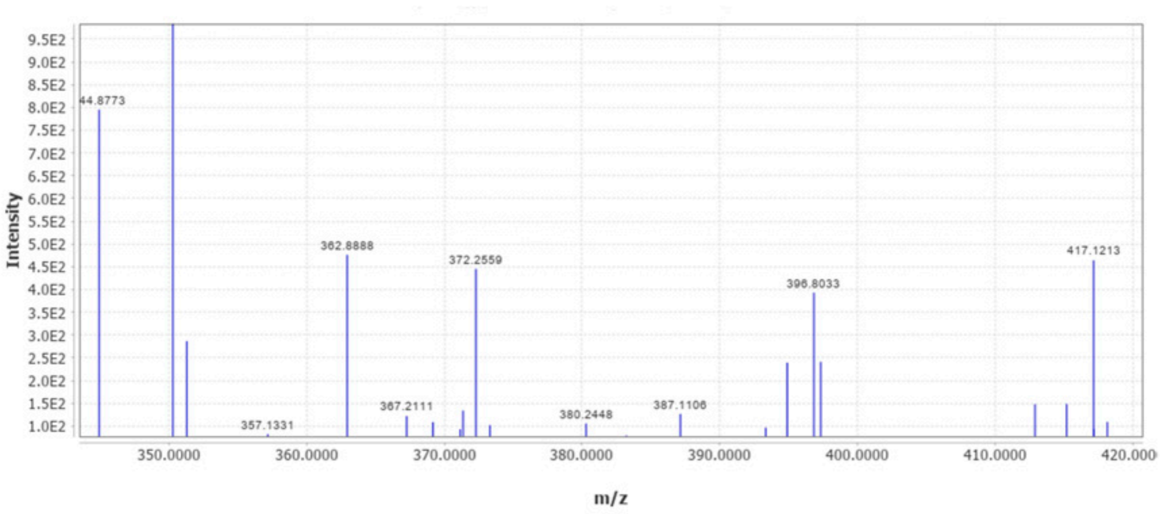

Figure S19: MS/MS spectrum of Puerarin (Peak 17)

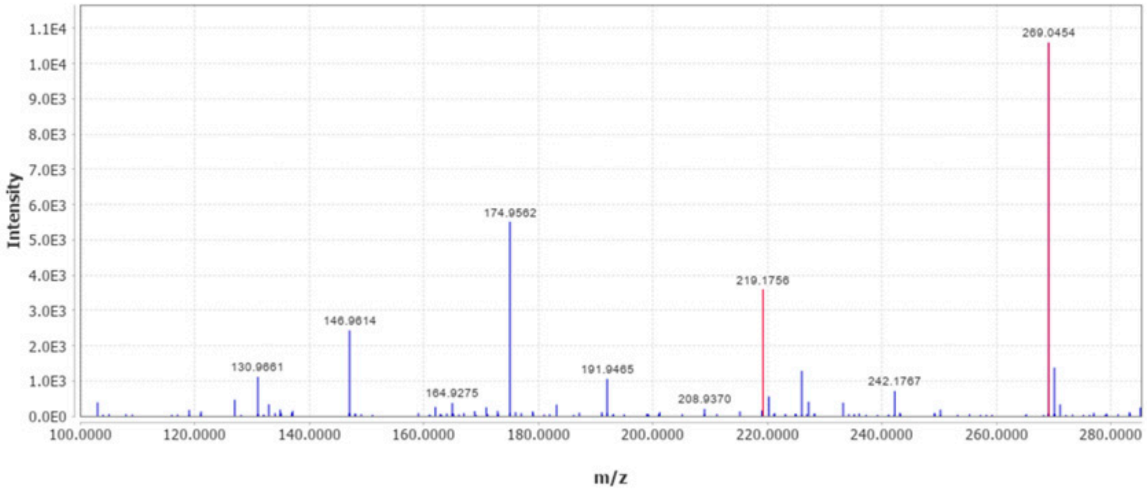

Figure S20: MS/MS spectrum of Genistein (Peak 18)

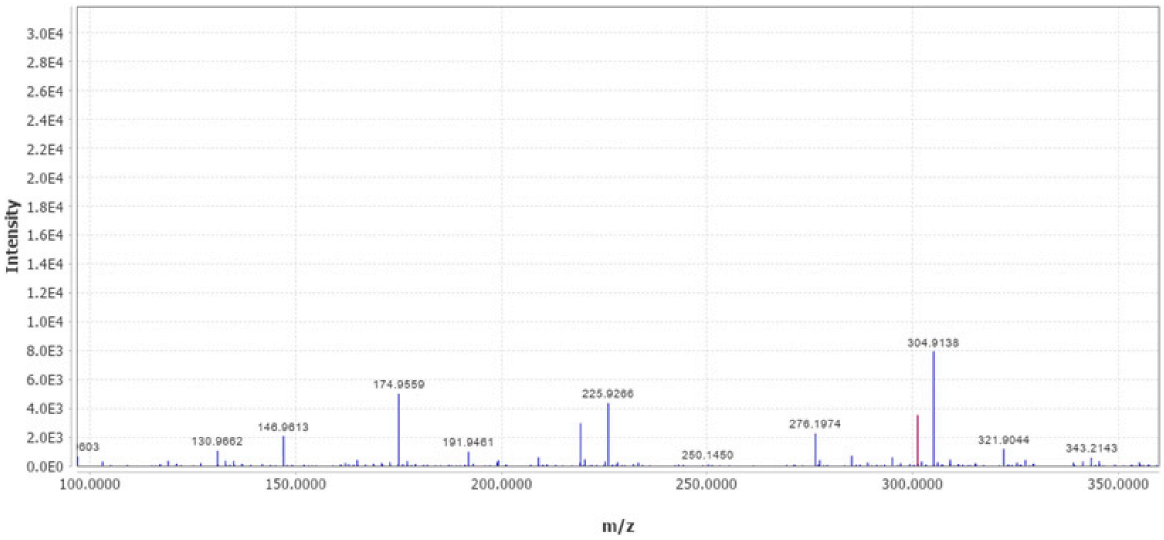

Figure S21: MS/MS spectrum of Quercetin (Peak 19)

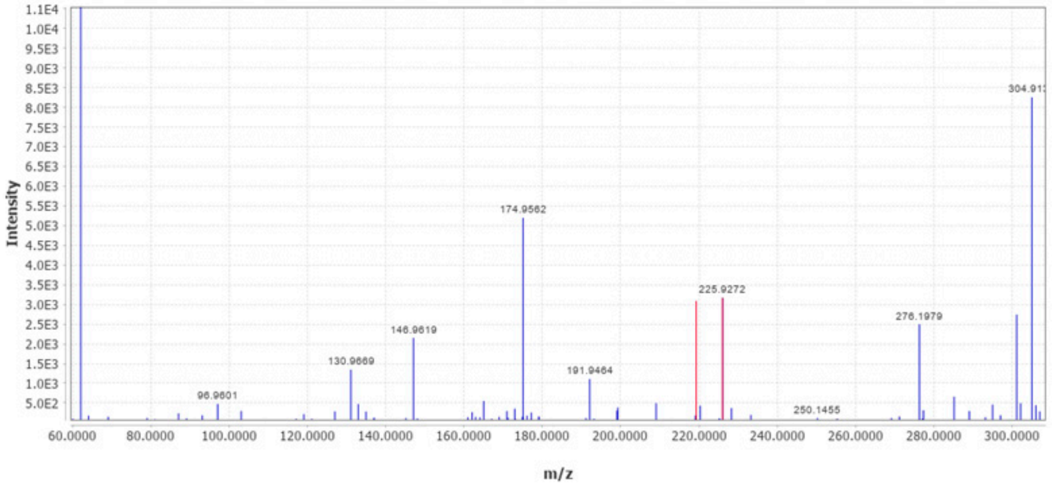

Figure S22: MS/MS spectrum of Luteolin (Peak 20)

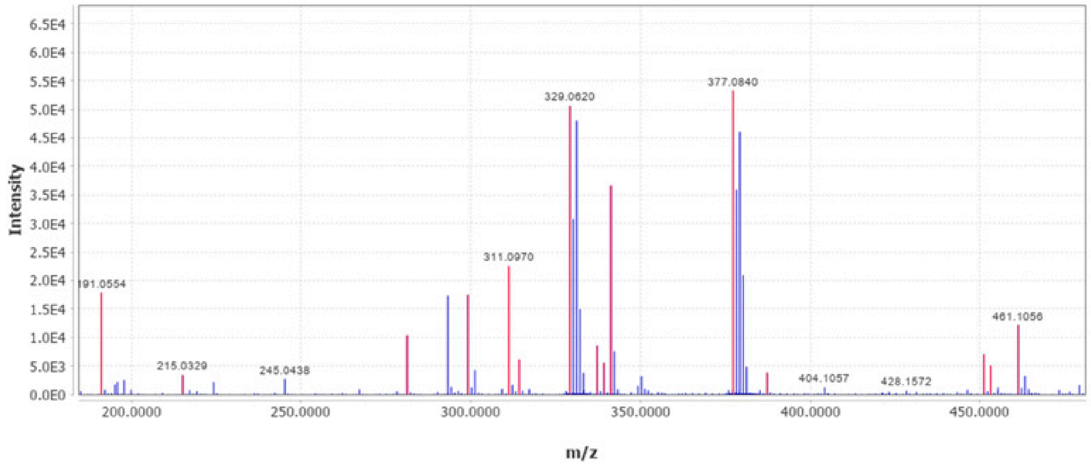

Figure S23: MS/MS spectrum of Tectoridin (Peak 21)

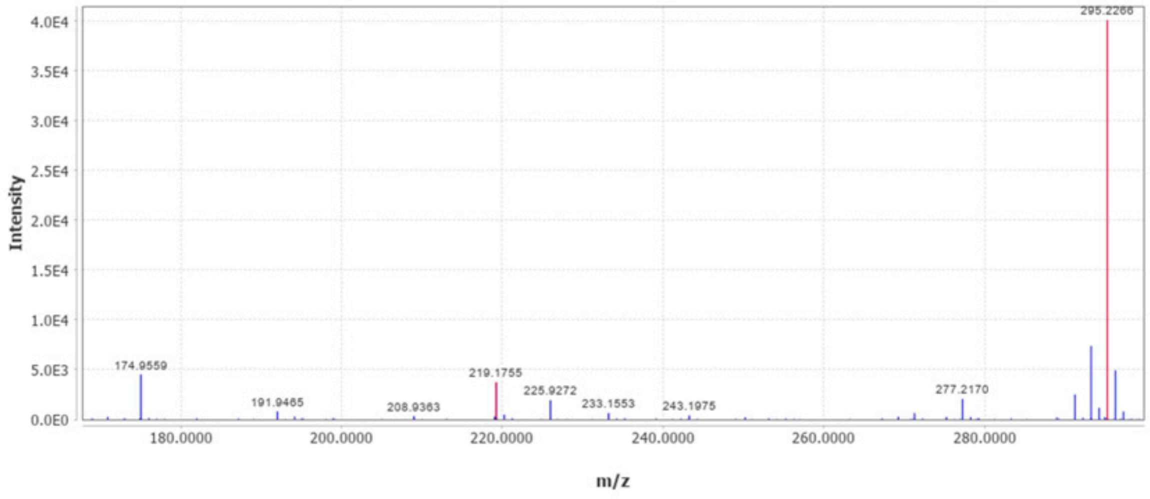

Figure S24: MS/MS spectrum of hydroxy octadecadienoic acid (Peak 22)

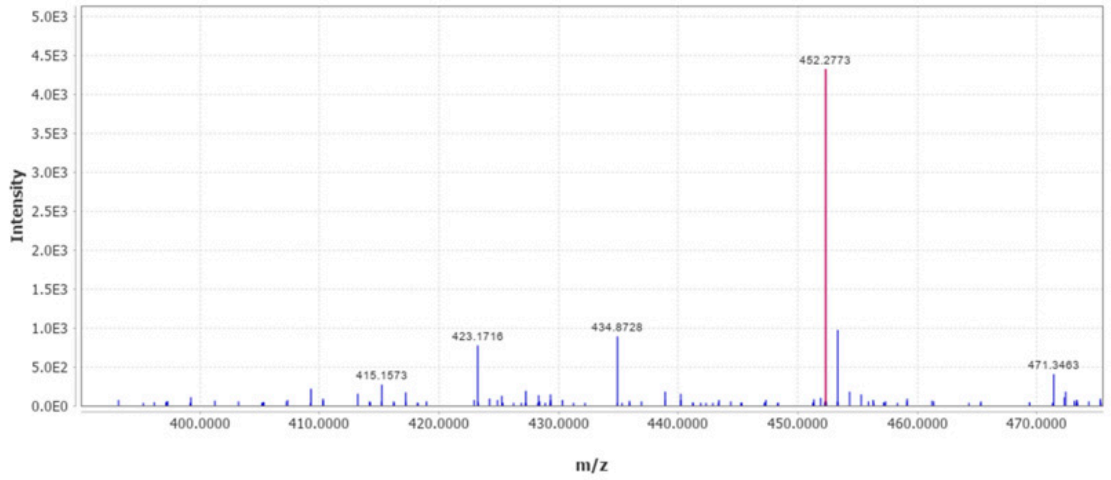

Figure S25: MS/MS spectrum of Hederagenin (Peak 23)

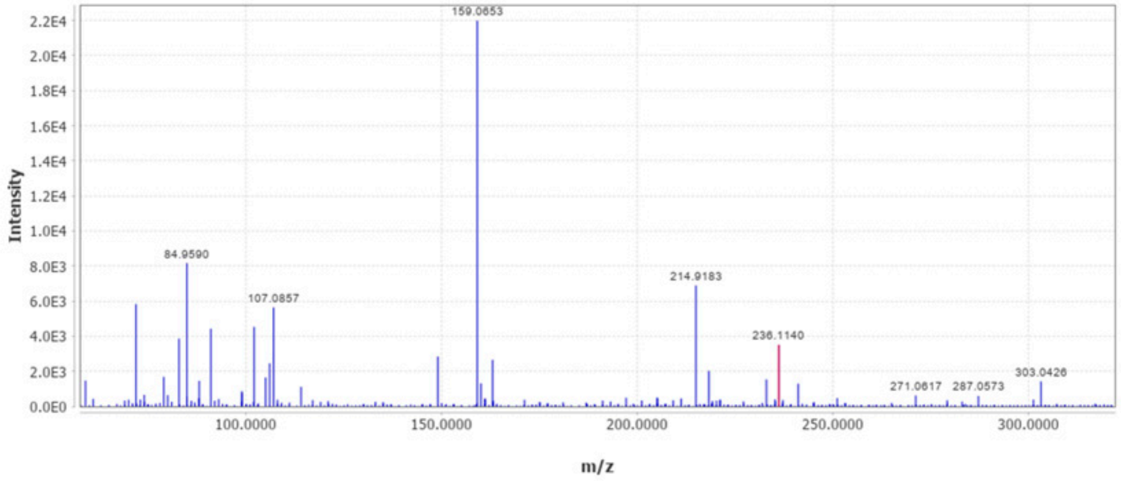

Figure S26: MS/MS spectrum Acacetin (Peak 24)

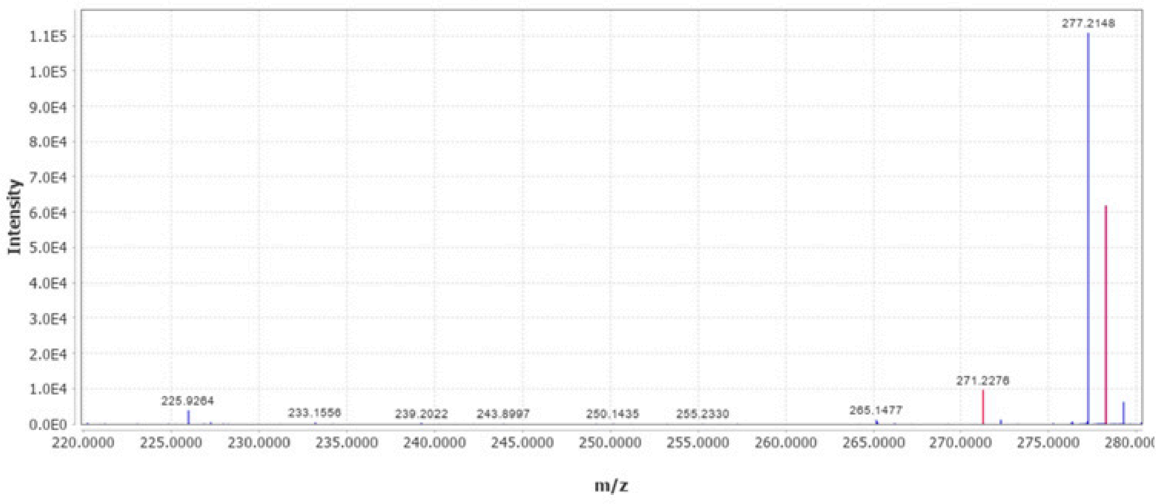

Figure S27: MS/MS spectrum of Hydroxy palmitic acid (Peak 25)

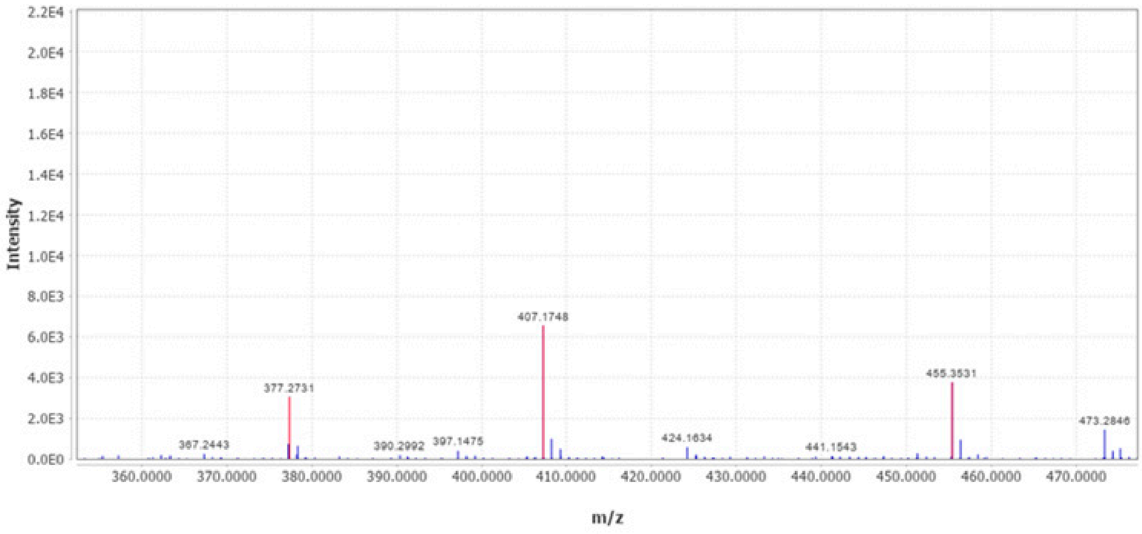

Figure S28: MS/MS spectrum of Oleanolic acid (Peak 26)

	**Control**	**2000 mg/kg**	**5000 mg/kg**
**Initial weight (g)**	192 ± 11.2	189 ± 17.8	180 ± 14.9
**Final weight (g)**	212 ± 10.6	204 ± 18.9	197 ± 17.4
**Body weight gain (g)**	20.4 ± 5.81	14.8 ± 2.28	17.2 ± 3.96
**Body weight gain (%)**	10.7 ± 3.23	7.85 ± 1.15	9.53 ± 1.92

Table S1: Effect of CEE on body weight changes

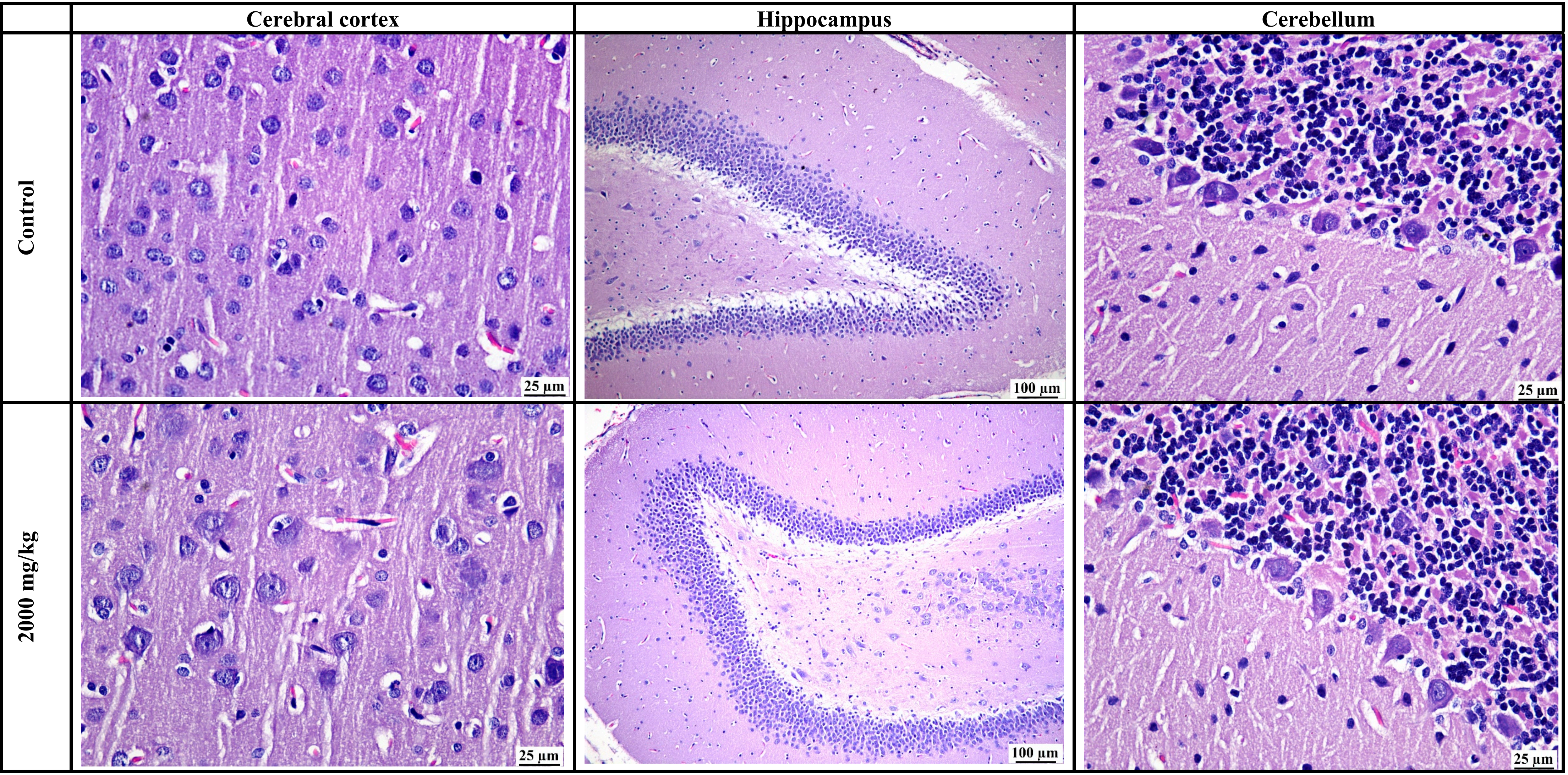

Cerebral cortex
Hippocampus
Cerebellum
Control
2000 mg/kg
25 µm
100 µm
25 µm
25 µm
100 µm
25 µm

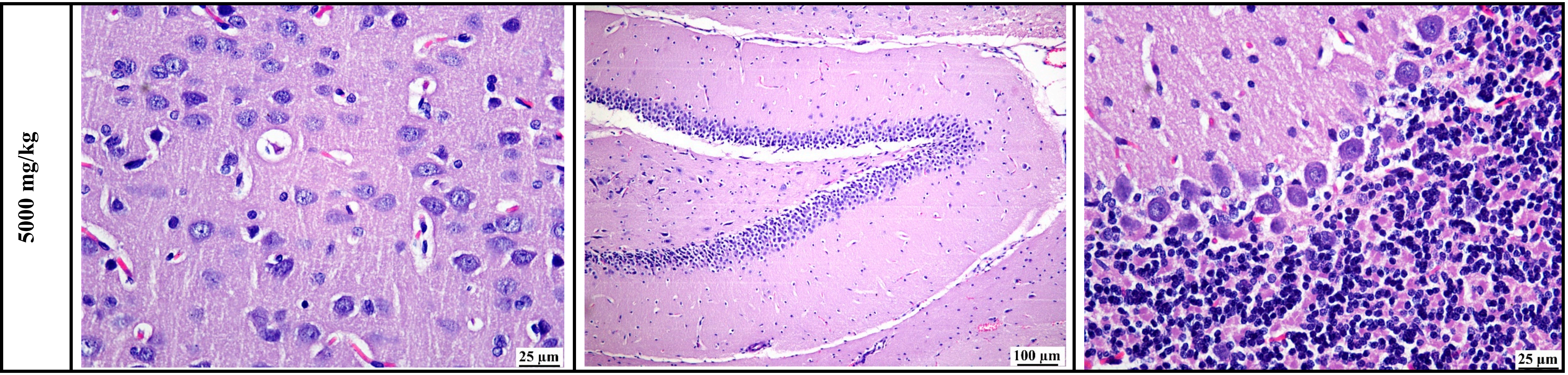
5000 mg/kg
25 µm
100 µm
25 µm

	200X	400X
Control	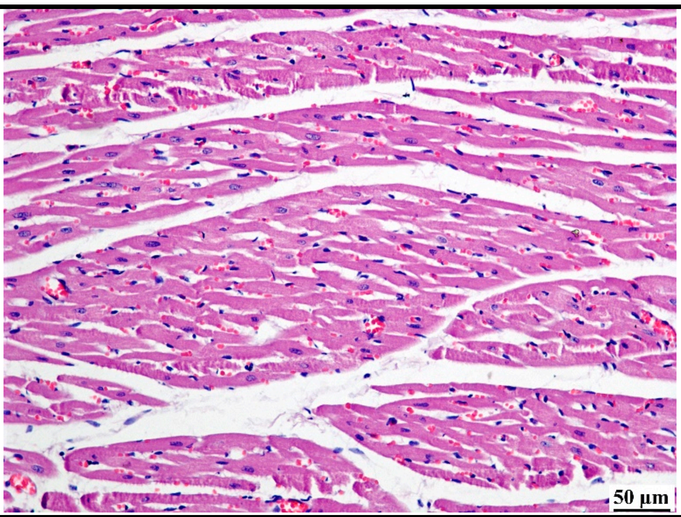	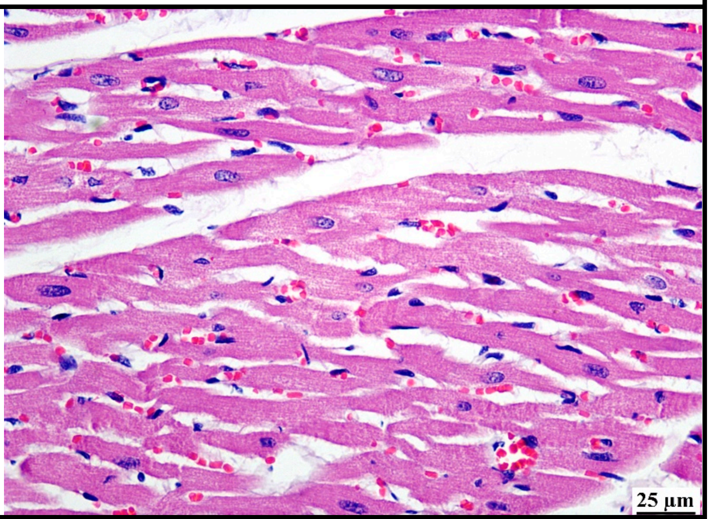
2000 mg/kg	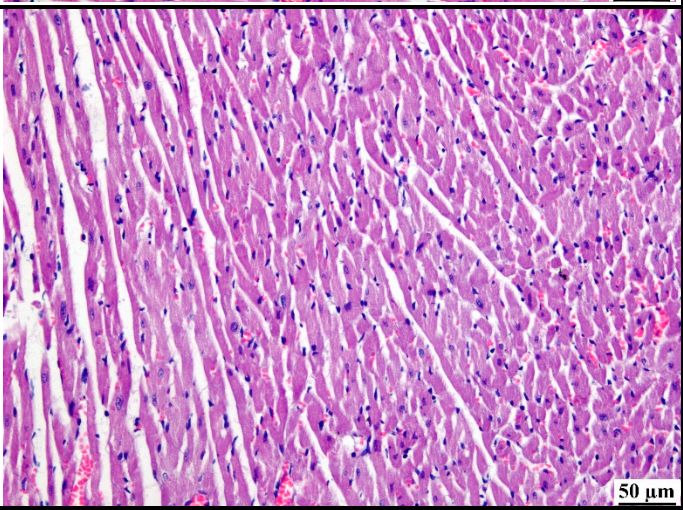	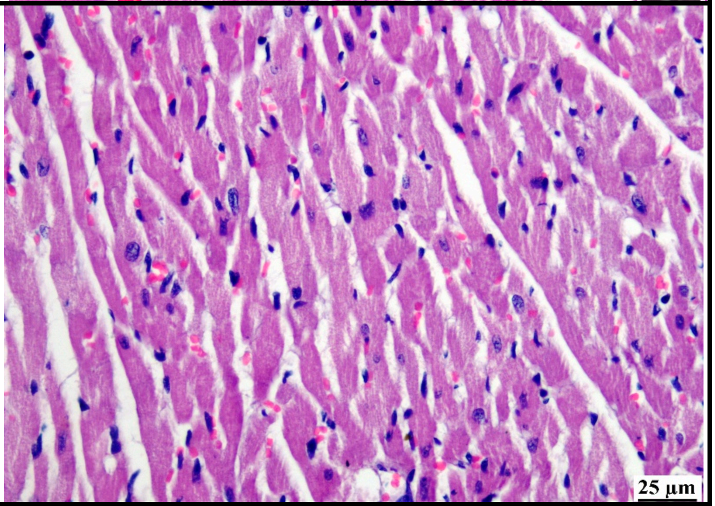
5000 mg/kg	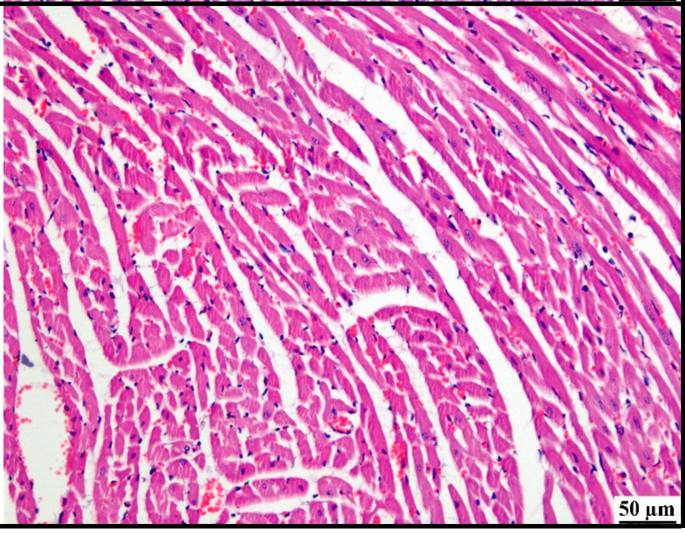	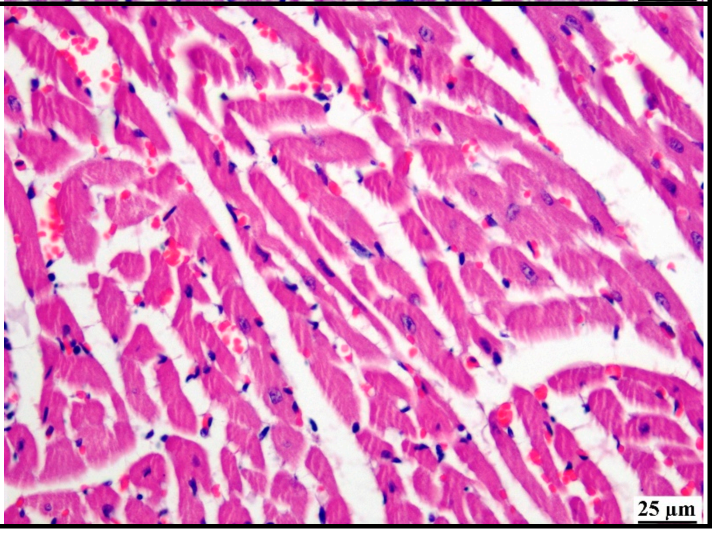

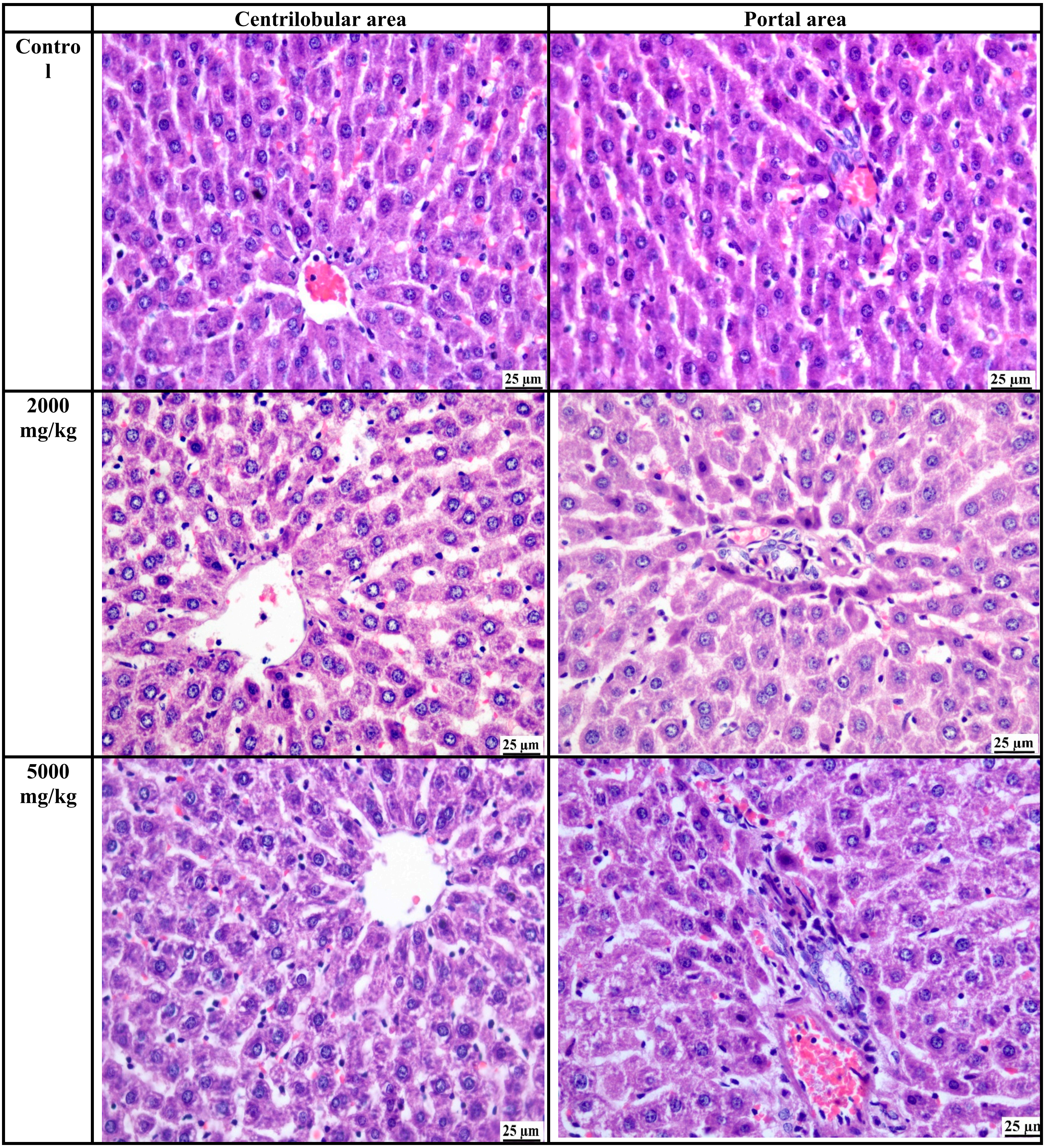
Centrilobular area
Portal area
Contro
l
2000
mg/kg
5000
mg/kg
25 µm

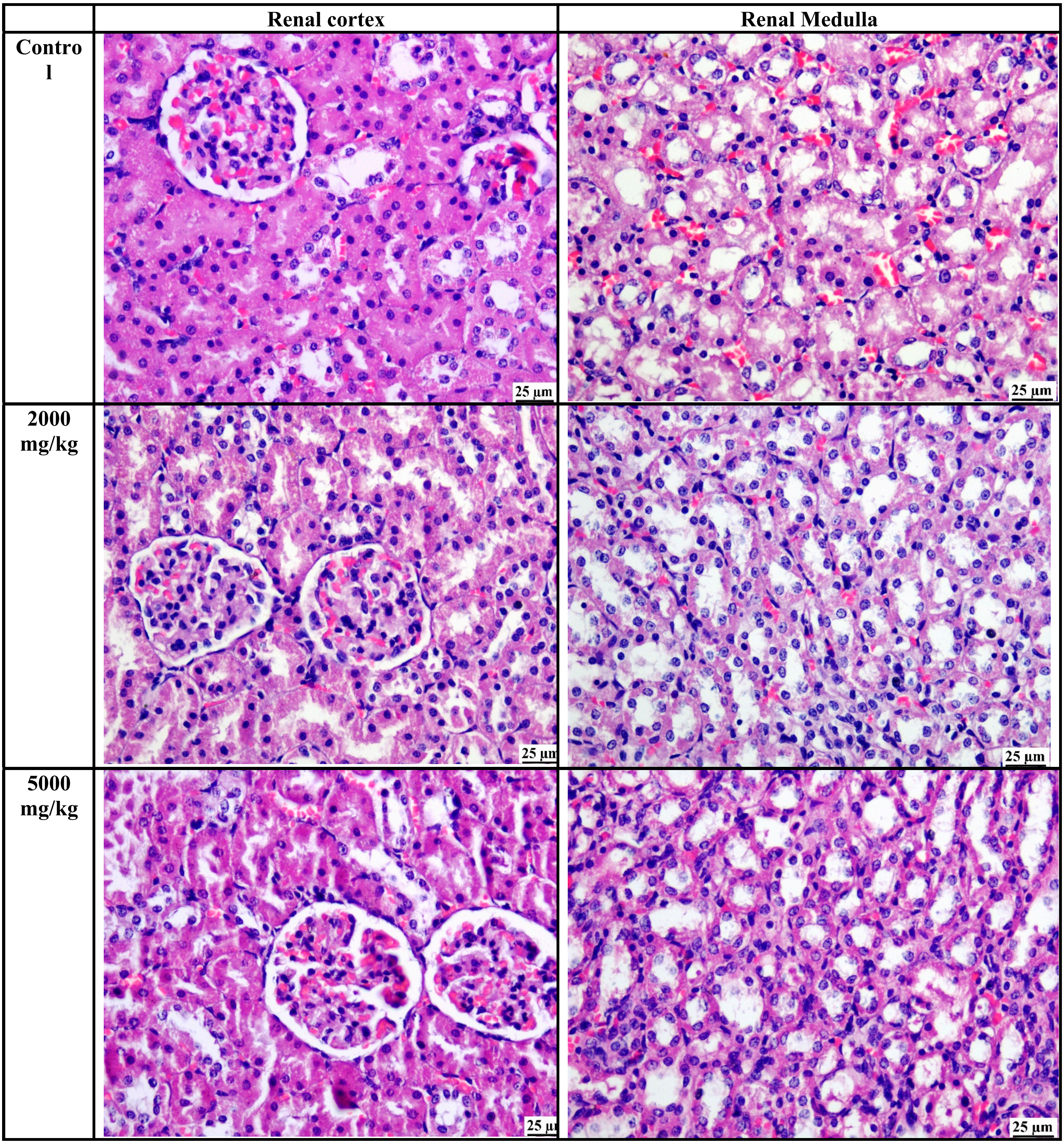

Figure S29: Microscopic examination of different brain regions, heart, liver, and kidney
